# Motor constellation theory: A model of infants’ phonological development

**DOI:** 10.3389/fpsyg.2022.996894

**Published:** 2022-11-03

**Authors:** Axel G. Ekström

**Affiliations:** Speech, Music and Hearing, KTH Royal Institute of Technology, Stockholm, Sweden

**Keywords:** phonological development, biology of speech, child development, reinforcement learning, neurolinguistics, speech acquisition

## Abstract

Every normally developing human infant solves the difficult problem of mapping their native-language phonology, but the neural mechanisms underpinning this behavior remain poorly understood. Here, motor constellation theory, an integrative neurophonological model, is presented, with the goal of explicating this issue. It is assumed that infants’ motor-auditory phonological mapping takes place through infants’ orosensory “reaching” for phonological elements observed in the language-specific ambient phonology, *via* reference to kinesthetic feedback from motor systems (e.g., articulators), and auditory feedback from resulting speech and speech-like sounds. Attempts are regulated by basal ganglion–cerebellar speech neural circuitry, and successful attempts at reproduction are enforced through dopaminergic signaling. Early in life, the pace of anatomical development constrains mapping such that complete language-specific phonological mapping is prohibited by infants’ undeveloped supralaryngeal vocal tract and undescended larynx; constraints gradually dissolve with age, enabling adult phonology. Where appropriate, reference is made to findings from animal and clinical models. Some implications for future modeling and simulation efforts, as well as clinical settings, are also discussed.

## Introduction

Human infants are born into complex phonological landscapes, composed of a set of a near-infinite number of possible speech sounds (Maddieson, 1984). At birth, human infants possess a limited vocal repertoire, including crying and moaning (Eibl-Eibesfeldt, 1973; Ackermann and Ziegler, 2010). From such humble beginnings, they display predictable linguistic development across individuals, languages, and cultures, adapting to and acquiring almost flawlessly their native language and phonology (here operationalized as any language-specific set of permissible speech sounds). In under a year, every normally developing infant learns to reliably perceive the sounds of his or her native language (Werker and Tees, 1984; Kuhl et al., 1992; Cheour et al., 1998), and has begun consistently producing language-appropriate syllabic utterances in the form of babble and vocal play (Locke and Pearson, 1992; Guenther, 1994, 1995; Oller, 2000; Jang et al., 2019). The remarkable speed of this development has been the subject of decades of intense research efforts (Oller, 1980, 2000; Jusczyk, 1997; de Boysson-Bardies, 2001). Infant cries, once believed a possible precursor of speech (Lester and Boukydis, in press), are no longer considered as such (Nathani et al., 2006; Oller et al., 2013, 2021). Rather, protophones, infant speech-like utterances including vowel-like sounds and melodic non-cry vocalizations, appearing even before the onset of babble, represent a substantially greater proportion of infant utterances (Stark, 1980; Hsu et al., 2000; Jang et al., 2019; Oller et al., 2021; Wermke et al., 2021) and are considered likely precursors of phonemes proper (Oller, 1980; Koopmans-van Beinum and Stelt, 1986).

At around 6 months of age, infants begin producing canonical babble—repetitions of the same syllable, e.g., /ˈbɑːbɑː/—and around the age of 1 year, begin producing variegated babble—more complex mixed-syllable utterances, e.g., /ˈbɑdə/ (Oller, 2000). Crucially, adequate learning of phonological patterns may facilitate learning of other aspects of language (for a review, see Ruben, 1997). While the vocal milestones reached throughout infanthood have been alternately described by multiple researchers and using varied terminology (reviewed in Vihman, 2013), these general trends and tendencies are not controversial in the literature. Nevertheless, the mechanisms by which infants manage this mapping of language-appropriate sounds to their corresponding points of articulation are poorly understood.

Humans are vocal learners (Janik and Slater, 2000), capable of memorizing and repeating vocally that which has previously been heard. Indeed, human infants exhibit variable generalized imitative behavior with likely bearing on later-in-life speech behavior, including the imitation of facial expressions (Field et al., 1982, 1983), gestures such as tongue protrusion and head movements (Meltzoff and Moore, 1989), as well as goal-directed physical actions (for a review, see Elsner, 2007) and vocalization more broadly (Poulson et al., 1991; Kuhl and Meltzoff, 1996; Kugiumutzakis, 1999; Kokkinaki and Kugiumutzakis, 2000). Neural mechanisms underlying imitation are not yet well understood, but Marshall and Meltzoff (2014) have pointed to mirror neurons—cells triggered upon both the execution of an act, and the observation of the same act (de Di Pellegrino et al., 1992)—as a possible explanation. In terms of behavioral measures, Imafuku and colleagues found that infants’ tendency to vocally imitate vowel sounds was based both on infants’ attention to speakers’ faces, and whether a speaker’s gaze was focused on the infant in return (as opposed to away from the infant; Imafuku et al., 2019).

Human neonates, seemingly based on prosodic and indexical cues, prefer the sound of their mother’s voice, heard *in utero*, as well as the sounds of their mother’s language (Jusczyk et al., 1993; see overview in Locke and Snow, 2010). Thus, systems of perception undergo a process of adapting to ambient phonological features, beginning even before birth. Phonetically, however, the tuning of systems of speech production to match a native-language phonology represents a monumental task (for a comparative perspective, see Bolhuis, 1991), and the history of the field has seen a range of theories with bearing on the phenomenon, from “innatist” theories assuming a hard-wired cognitive apparatus prepared for learning speech and language (Chomsky, 1986, 2002), to modern input-focused theories, assuming development scaffolding through infants’ interactions with caretakers (Fernald, 1991; Kuhl et al., 1997; Goldstein and Schwade, 2008) or, more generally, acquisition based on learning from the immediate environment (including parental speech; Kuhl, 2000; Perszyk and Waxman, 2019). Supporting evidence is also available from computational modeling and learning approaches (Vallabha et al., 2007).

Despite the range of theories, however, much remains unknown about the mechanisms that underlie infants’ language development. While innatist accounts have been criticized for evolutionary implausibility (Pinker and Bloom, 1990), interactionist theories have found significant support in relevant research (Poulson et al., 1991; Kuhl and Meltzoff, 1996; see review by Chapman, 2000). However, such accounts suffer on theoretical grounds, being heavily based on observation (see Chapman, 2000; Lindblom, 2000). In the words of Chapman (2000, 33), the field has “been productive in identifying developmental patterns and individual differences but slow to develop explanations that are more than a relabeling of the patterns observed.”

Some basic postulates for a theory of phonology as an emergent phenomenon have been presented by Lindblom (2000). Namely, a theory of infants’ phonological learning must—as opposed to “curve-fitting,” the tailoring of explanatory models based solely on observations—be predicated on basic principles of the natural world, while also accommodating empirical findings. The present account accepts this premise, and thus seeks to consider both the deeper biomechanical origins and necessarily pre-verbal development and subsequent employment of in-place motor activity in early speech-like behavior (Lindblom, 2000; MacNeilage and Davis, 2000); that is, principles of learning by which a system of phonology develops from non-systematic exploratory pre-speech; and the neurological changes that accompany these developments. A theory seeking to explicate such a complex and ultimately neuroscientific issue must couch its propositions in a more basic body of literature from the study of learning, phonetics, developmental psychology, and comparative cognition and neuroscience. Providing such a framework is the goal of the present text.

In the following sections, the basics of speech production, and the neural activity to which it corresponds, are reviewed. Drawing on comparative research, including clinical observations and findings from animal models, a theory of phonological development is presented. It is suggested that dopaminergic pathways in the infant brain instantiate learning of tutor (i.e., parent or other ingroup caretaker) phonology, by comparing auditory outputs resulting from a given motor constellation (i.e., simultaneous activation of muscle groups) to target goals, derived from ingroup ambient input. This process is presumed guided *via* reference to kinesthetic and auditory feedback. Key assumptions are summarized in a theoretical framework, with some tentative implications for modeling approaches and clinical work. Said framework is dubbed the motor constellation theory of infants’ phonological development.

## Navigating phonetic output

### Speech production and acoustics

Human speech is a behavioral composite of motor activity in the respiratory organs, larynx, and articulatory organs—the tongue, upper and lower lips, upper teeth, alveolar ridge, hard palate, velum, uvula, pharyngeal wall, and glottis—executed in combination (for overviews, see Denes and Pinson, 1963; Ladefoged, 1996; Stevens, 2000). Speech production results from air being expounded from the lungs at variable pressures, causing vibration in the vocal folds of the larynx (except in, e.g., whispering, where vocal folds do not vibrate), and air pressure is forced through structures in the vocal tract imposing narrow constrictions on airflow (Denes and Pinson, 1963). The rate of vocal fold vibration is termed the fundamental frequency (*f*_0_) and corresponds perceptually to pitch height, while the imposition of narrow constrictions results in variations (mainly) in the first and second formants (F_1_ and F_2_, respectively)—spectral frequency peaks resulting from resonances in the vocal tract—where F_1_ is predominantly determined by the height of the tongue body, and inversely related to vowel height, such that lower frequencies correspond to greater vowel heights; and F_2_ largely determined by tongue front-to-back position, corresponding to the frontness/backness of a vowel. All spoken languages, thus, share a most basic property, that of being composed of culturally agreed-upon (though largely arbitrary) formalized constellations of motor activity, cognitively imbued with symbolism (i.e., word semantics).

The number of vowels, consonants, and phonemes in a given language is highly variable (Maddieson, 1984), but never exhausts the full potential rendered possible by human systems of speech production. The phonetic structure of vowel systems—that is, the qualities of vowels sustained as part of a language-specific phonology—is contingent on perceptual contrast between vowels (Lindblom and Sundberg, 1969; Liljencrants and Lindblom, 1972). Results of early modeling by Lindblom and Sundberg (1969) investigating the maximum distance between permissible vowels within a random set (while still allowing for intelligibility and sufficient distinctiveness) further point to a role for limitations of perception and memory in the construction and maintenance of language-specific phonologies. Similar principles also govern the structure and development of consonant systems (Lindblom and Maddieson, 1988). It need not be argued that a language–and its associated system of speech sounds–must be simple enough to be perceived and repeated by infants born into the society that speaks it; any language that did not abide by this principle would fail to survive beyond a single generation of speakers. Thus, systems of speech must be flexible enough to allow for the variant qualities, inherent both in the speech signal itself, and in the perceptual systems of listeners. What is built up by the infant in acquiring phonology, then, is a library of systematic knowledge of the relationship between auditory patterns, kinesthetic-orosensory patterns, and (for purposes of modeling) discrete target positions (Fry, 1966; Lindblom and Sundberg, 1969; Boysson-Bardies et al., 1992).

### Developmental constraints on infants’ phonological production

Phonological mapping must necessarily be limited by constraints of the developing vocal apparatus (Green and Nip, 2010); for example, the anatomical prerequisites for the production of nasal bilabials such as /m/ or fricative bilabials such as /b/ are largely present at birth, leading to typically observed first words (roughly corresponding to, e.g., /ˈbɑːbɑː/, /ˈmɑːmɑː/; McCarthy, 1946). Meanwhile, fricative alveolars such as /s/ require significant lingual muscle dexterity (not to mention dentition) before its cognitive-orosensory coordinates can be appropriately mapped and accommodated. The same is also true of vowel sounds. For example, utterances such as schwa (in English, an unstressed, or neutral vowel) require comparably little effort or flexibility on behalf of a speaker, compared to, e.g., /i/, which requires significant labial and lingual stretching, as well as the development of necessary anatomical interstructural relationships. In adult humans, roughly half the tongue is positioned in the throat, such that the supralaryngeal airway acquires a roughly right-angle bend at its midpoint. The resulting near 1:1 relationship between horizontal and vertical sections of the supralaryngeal vocal tract (SVT) renders possible the production of quantal vowels /a/, /i/, and /u/ (Stevens, 1972, 1989). However, the same relationship is not found in infants.

Instead, at birth, the tongue is largely contained in the mouth, only descending into the throat with development, reaching completion by roughly 8 years of age (Lieberman, 2012). As the tongue descends, so does the larynx, which is also positioned higher in infants compared with adults (Lieberman et al., 2001; Nishimura, 2018). With SVTs more similar to those of nonhuman primates than of adult humans, human infant SVTs are incapable of producing quantal vowels (Lieberman et al., 1972; Stevens, 1972, 1989; Lieberman, 2012), and their corresponding mapping thus cannot be completed prior to this point of development. That is, the maturing SVT provides increased proprioceptive-auditory affordances (see Gibson, 1979), as exploration of its motor and acoustic-perceptual relationships becomes available. Accordingly, infants’ vowel space (Kent and Murray, 1982), utterance melodic complexity (Wermke et al., 2021), and (in infants acquiring a tonal language) accuracy of tonal suprasegmental features as well as the complexity of individual tones readily acquired (Wong and Strange, 2017)^1^ all increase significantly throughout the first year of life with the development of increased lingual and muscle dexterity and flexibility. Such contingence on anatomy places significant constraints on the infants’ initial phonetic development.

### Articulation is position control

Even in the most mundane everyday activities such as reaching for an object or placing one foot in front of the other, human actors make use of sophisticated computation when acting upon the world. Neurologically, such instances of fine position control are continually adjusted by cerebellar-motor cortex networks (Drew, 1993; Armstrong and Marple-Horvat, 1996; Drew et al., 2008), *via* reference to both visual feedback from the immediate environment, and proprioceptive-kinesthetic feedback from relevant muscle groups. Necessary adjustments to fine-motor movements are readily accomplished with little or no premeditation; this phenomenon is termed motor equivalence—the use of variable motor sequences of muscle movements toward achieving some goal. However, the broad domain-general functionality of cerebellar networks for motor control extends beyond reaching, grabbing, and walking. Indeed, there is significant evidence of motor equivalence in speech articulation also. Findings presented by Gay and colleagues on compensation in vowel production in conditions of abnormal jaw openings (Lindblom et al., 1979) and bite blocks (Gay et al., 1981) suggest (1) that articulation is compensatory and (2) that tongue placement is executed appropriately *via* reference to tactile feedback.

**Figure 1:**
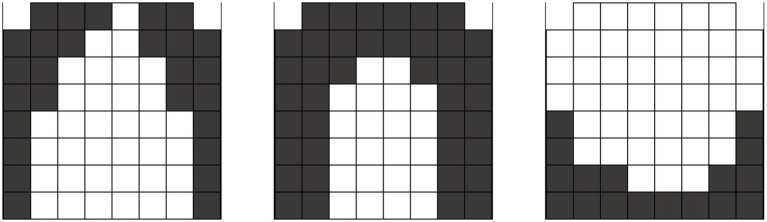
Tongue contact patterns for consonantal sounds. Left to right: alveolar grooved /s/ /z/; alveolar stop /t/ /d/ /n/; velar stop /k/ /g/ /ng/.

The human tongue possesses four major extrinsic muscles: (1) the genioglossus, which extends, protrudes, and depresses the tongue; (2) the styloglossi, which retract the tongue; (3) the hyoglossus, which depresses and retracts the tongue; and (4) the palatoglossus, which elevates the posterior position of the tongue, and four intrinsic (attaching only to other muscles in the tongue body) paired muscles, the (1) superior longitudinal and (2) inferior longitudinal and (3) transverse and (4) vertical muscles, whose directions of travel are all indicated by their nomenclature. Each muscle or group of muscles is dominant to others in given contract patterns (see Figure 1). Further bridging the gap to motor equivalence in reaching, Moayedi et al. (2021, 3046) have recently suggested that “the organization of [tongue] somatosensory endings is reminiscent of fingertips, suggesting that the hard palate is equipped with a rich repertoire of sensory neurons for pressure sensing and spatial localization of mechanical inputs.” Thus, speech articulation may be defined as the “reaching” in laryngeal–orosensory space for discrete target positions, defined, in turn, as contact patterns.

However, muscles of the tongue are merely one example of sources of feedback necessary for appropriate articulation. Significant evidence now also points to the role of multimodal feedback in the control of speech articulatory and acoustic parameters, the first and most obvious being auditory feedback.

### The role of feedback

Evidence for the necessity of auditory feedback in speech articulation is provided by a range of experiments wherein that feedback is perturbed, and production is adjusted to compensate. Effects of perturbing the auditory feedback channel can be examined by applying real-time frequency modulation of speaker voice (Elman, 1981; Kawahara, 1994). Results of such studies typically observe that subjects shift *f*_0_ in the direction opposite that of the stimuli presented (Burnett et al., 1998; Jones and Munhall, 2005; Larson et al., 2008), but other perturbation experiments have also observed compensatory shifts in F_1_ and F_2_ (Houde and Jordan, 1998; Purcell and Munhall, 2006; Pile et al., 2007; Katseff et al., 2012). Compensation to perturbation takes place within 150 ms of perturbation onset, and mismatches are coded bilaterally in the superior temporal cortex of the speaker (Tourville et al., 2008). Beyond auditory feedback, the laryngeal mucosa sensing vibrations in the laryngeal cavity (during vocal fold oscillation) also provide important somatosensory feedback. That is, vibrotactile feedback stemming from activity directly in the larynx may also serve as a clue to whether desired vocal production is in fact being executed (see also Shiba et al., 1997; Sapir et al., 2000). As noted by Hammer and Krueger (2014), who tested laryngeal mechanosensory detection thresholds using endoscopy, the sensorium of the larynx itself also appears to modulate afference, attenuating potentially distracting sensory input mid-vocalization.

Indeed, available evidence now suggests that control of articulation is supported by dual feedback channels of auditory and proprioceptive feedback. Work by Schroeder and colleagues examining recordings of macaque monkey (*Macaca mulatta* and *M. fascicularis*) auditory association cortices, when subjects were presented with auditory and somatosensory input, suggest a significant temporal overlap between the two, as well as integration at an early stage of auditory cortical processing (Schroeder et al., 2001). Wang and colleagues investigated the simultaneous influence of auditory and vibrotactile feedback disturbances in *f*_0_ control in human subjects, finding stronger compensatory responses in participants in a combined vibrotactile-auditory stimuli condition than for either single modality on its own (Wang et al., 2015a,b; see also Larson et al., 2008).

Such findings are complemented by work by Katseff et al. (2012), who upon finding that subjects compensated more for small feedback shifts than for larger ones, suggested that auditory and somatosensory information was incorporated by a speech motor control system, apparently driven by differential weighting of both modality parameters: Where discrepancies are minor, a premium may be placed on auditory feedback, while for greater discrepancies, somatosensory feedback may outweigh auditory feedback (Katseff et al., 2012). Reflecting the role of both auditory and proprioceptive feedback, feedback parameters are included, as a means of articulatory correction, in speech motor control modeling efforts such as Frank Guenther’s DIVA model (Guenther, 1995; Guenther and Vladusich, 2012). Significantly for the present account, Locke (1993) has also stressed similar roles of feedback for facilitating development of speech capacities in the human child. Indeed, when learning a new motor skill (including the production of any phoneme or set of phonemes), sensory feedback provides crucial referent information; any physical action corresponds to a unique proprioceptive-kinesthetic perceptual experience, which in learning that skill helps facilitate its repetition (e.g., Ullman, 2001).

## From perception to production

While intraspecies social vocalization represents an ancient evolutionary heritage (Bass et al., 2008), vocal learning is an ability shared with only a few disparate lineages, including pinnipeds (Schusterman, 2008; Reichmuth and Casey, 2014), bats (Vernes and Wilkinson, 2020), and cetaceans, such as whales (Noad et al., 2000) among mammals; and parrots (Pepperberg, 2010; Bradbury and Balsby, 2016), hummingbirds (Baptista and Schuchmann, 1990), and oscines (hereafter songbirds) among Aves. Among primates, only humans consistently exhibit sophisticated vocal learning (Egnor and Hauser, 2004; but see, e.g., Wich et al., 2009). Of all vocal learning capacities currently known to science, the human ability is rivaled in complexity only by songbirds. Further, outside of humans, songbirds represent by far the most well-studied vocal learning taxonomic group (Konishi, 1964, 1985, 2010; Nottebohm, 1970; Marler and Waser, 1977; Nottebohm et al., 1986; Kroodsma and Konishi, 1991; Bolhuis and Gahr, 2006; Bolhuis et al., 2010; Gale and Perkel, 2010; Bolhuis and Moorman, 2015; Prather et al., 2017).

Though features of songbird vocal anatomy and physiology (Greenwalt, 1968; Suthers, 1997) differ from those of humans (e.g., Ladefoged, 1996) and nonhuman mammals (Negus, 1949; Harrison, 1995)—and though such differences lead to obvious differences in acoustic output—the two systems can be usefully thought of as comparable. Systems of vocalization in both species are *a priori* free (there should be no objectively more beneficial system of vocalization) and subject to relatively well-defined constraints, including the limitations resulting from the progressive development of the speech apparatus of humans (Lieberman et al., 1972; Green and Nip, 2010; Lieberman, 2012), and song apparatus of songbirds (Greenwalt, 1968; Farries, 2004). There are also remarkable similarities between songbird and human brains, resulting from convergent evolution (Colquitt et al., 2021). Thus, over the course of the development of the field, multiple authors have drawn on the behavioral parallels between birdsong and human speech (Marler, 1970; Doupe and Kuhl, 1999; Goldstein et al., 2003; Kuhl, 2003; Bolhuis et al., 2010; Prather et al., 2017) and such parallels have at times guided the interpretation of experimental work on linguistic development (e.g., Goldstein et al., 2003).

In any species capable of vocal learning, developing individuals must solve a difficult adaptive problem in ontogeny, that is, adapting one’s repertoire of vocal output to ambient sounds as observed in mature conspecifics. In songbird species such as the Zebra finch (*Taeniopygia guttata*), auditory feedback is necessary for matching explorative vocal output against intended sounds. This was most clearly made evident through the work of Masakazu Konishi in his studies of deafened songbirds, that failed to develop adequate song (Konishi, 1964, 1965b; see also Marler and Waser, 1977; Price, 1979; Brainard and Doupe, 2000). Similarly, deaf-born human infants exhibit impaired development of babbling behavior (Oller and Eilers, 1988) and later in life typically present with underarticulated (e.g., Hudgins and Numbers, 1942) and monotone (e.g., Smith, 1975) speech. Unlike songbirds, suboscines such as chickens (*Gallus domesticus*) produce species-typical vocalizations, even when deafened (Konishi, 1963a). In the case of species-typical learned vocalization behavior, thus, complex motor learning (underlying vocal learning) is contingent on sensory feedback, which guides the steering toward a target auditory output. Comparative findings in human infants have also been provided by Boysson-Bardies et al. (1992).

In his doctoral work, Konishi (1963b) posited “template theory,” according to which a juvenile songbird will memorize the song of a conspecific tutor individual, using that song as points of reference in future own song development and elaboration. A young bird hears its own song and compares it to that of its sensory template; in the event of a mismatch between the two, the bird continually adjusts its song until it matches the template. Konishi (1963b, 1965a) suggested that, in the process of song learning, a songbird converts an “auditory template,” derived from the song of adult tutor individuals, into a “proprioceptive template,” such that sensory feedback helps guide motor activity toward positional coordinates necessary to produce desired auditory outputs (see also Nottebohm, 1970). Modern research has shown light on some of the neural circuitry that underlies this apparent phenomenon. Namely, in the songbird brain, the caudomedial nidopallium is believed to be the site of auditory tutor song memory storage (Bolhuis and Gahr, 2006; Hahnloser and Kotowicz, 2010; Bolhuis and Moorman, 2015; Yanagihara and Yazaki-Sugiyama, 2016). A basal ganglion dopamine (DA) pathway appears to drive auditory preference and response, forming a neurological basis for song memory (Gale and Perkel, 2010; Barr et al., 2021; Daou and Margoliash, 2021).

For mammals, comparable auditory experience-dependent neuronal plasticity has also been observed in rodents (Sanes and Bao, 2009; de Villers-Sidani and Merzenich, 2011) but direct equivalent evidence for the neurological underpinnings of human infants’ phonological development is, to the knowledge of the author, as of yet not available. However, some evidence exists with apparent bearing on this issue. Crucially, Kuhl and Meltzoff (1996) documented how infants of only a few months of age produced vocalization resembling heard recorded vowels. Echoing template theory of Konishi (1963b), the authors suggested that infants derived perceptual representations of heard vocalizations, which are utilized as targets for subsequent speech production (Kuhl and Meltzoff, 1996). Indeed, research on cultural variations in infant crying and babbling strongly suggest that plasticity begins early in life. Newborns’ crying is influenced by ambient native-language prosodic cues (Mampe et al., 2009), which also influences later-in-life babble (De Boysson-Bardies et al., 1981; de Boysson-Bardies et al., 1984; de Boysson-Bardies et al., 1989; Levitt and Utman, 1992) and rhythmic-prosodic properties such as positionally appropriate syllabic lengthening (Levitt and Wang, 1991). Finally, reflecting the developing SVT, cultural variations in consonantal sounds may appear later in development, compared with vowels—which are comparatively easily produced—and exhibit early cultural influence (Chen and Kent, 2010; Lee et al., 2010; but see de Boysson-Bardies et al., 1989).

Kuhl et al. (2006) have shown that auditory experience drives a progressive process of integration of language-specific phonemes in auditory memory, which may be indicative of analogous neural circuitry to that observed in songbirds and rodents. Following this work, a parallel to birdsong template theory (Konishi, 1963b) has been put forward and elaborated by Kuhl and colleagues (Kuhl, 1992; Kuhl and Meltzoff, 1996; Kuhl et al., 2006; see also Vihman, 2019).^2^ Crucially, recent iterations of Frank Guenther’s DIVA model (Guenther and Vladusich, 2012; Guenther, 2016) present a coherent argument for how such conversion from auditory speech “chunk” component to motor vocal production behavior may take place; that is, two-way prediction of motor and sensory domains facilitates the establishment of a “speech sound map” (Guenther, 2016).

## Physiological bases of speech learning

### Neural representations

Investigations into somatosensory motor cortex representations of the speech organs and articulators go back to Wilder Penfield’s classic work on the cortical somatotopic mapping of—among others—the tongue, jaw, and lips (Penfield and Boldrey, 1937; Penfield, 1954). More recent work has localized the site of cortical control of the larynx, dubbed the laryngeal motor cortex (Brown et al., 2008, 2021; Simonyan and Horwitz, 2011; Dichter et al., 2018), as well as the site of overlap between larynx and jaw somatotopic representations (Brown et al., 2021; see also MacNeilage, 1998). The organization of the auditory cortical ventral and dorsal pathways of the brain also shows substantial interspecies similarity (Rauschecker and Scott, 2009; Rauschecker, 2012; Hage and Nieder, 2016). Notably, however, complex motor behaviors, including linguistic abilities, are contingent on distributed networks of circuitry, with various localized centers of activity (Mesulam, 1990; Lieberman et al., 1992). Syllabic articulation is thought emergent from constellations of coordinated activity in a constellation of representations of articulatory organs (Browman and Goldstein, 1989; Levelt, 1993; Guenther, 2006; Bouchard et al., 2013). For example, a dorsal pathway in the premotor and temporal cortices supports speech repetition (Friederici and Gierhan, 2013), and the “dual neural network model” posited by Hage and Nieder (2016) assumes that voluntary speech emerges individually *via* the development of a prefrontal cortical *volitional articulatory motor network*, that assumes control over a subcortical phylogenetically preserved *primary vocal motor network*.

While cortical representation of speech production is relatively well researched (Wildgruber et al., 1996; Gracco et al., 2005; Papoutsi et al., 2009), its subcortical underpinnings, now increasingly recognized as crucial to speech behavior, remain relatively poorly understood (Lieberman, 2000, 2012). Patients suffering damage to the basal ganglia (BG; a subcortical structure) often present with classic signs of Broca’s aphasia or Wernicke’s aphasia (i.e., impaired speech production and compression, respectively), even when Broca’s and Wernicke’s areas are left intact by stroke (Stuss et al., 1986; Alexander et al., 1987; overview in Lieberman and McCarthy, 2015). Further, Chrabaszcz et al. (2019) observed significant increases in high-gamma power activity in the subthalamic nucleus (as well as in the sensorimotor cortex) in Parkinsonian patients preparatory to speech production and persisting throughout articulation durations.

Intriguingly, basal ganglion circuitry so implicated also includes the ventromedial prefrontal cortex and Broca’s area—areas classically associated with the regulation of spoken language (Lieberman, 2000). Tellingly, Dronkers et al. (2007) have observed subcortical damage to the BG in Paul Broca’s classic case study, on the patient “Tan,” whose symptoms have traditionally been attributed to damage to Broca’s area (Brodmann areas 44,45; Broca, 1861). Patients presenting with damage to cortical but not subcortical areas may often recover from the injury (Alexander et al., 1987), whereas this is not true of patients presenting with damage to subcortical regions. Finally, various prefrontal cortical areas implicated in speech-centric behavior—including the medial and lateral premotor cortices—project to the BG (Alexander et al., 1987; Cummings, 1993; Guenther, 2006); various prefrontal regions have also been found to be sites of projection from the BG (Middleton and Strick, 2002), further cementing the importance of subcortical circuitry for speech-centric behavior. The related role of the cerebellum in human speech production, meanwhile, appears to be facilitation of temporal organization of speech into smooth rhythmic utterances, as well as prearticulatory organization; this has been outlined by Ackermann (2008).^3^

The rhythmic motor behavior underlying speech, finally, is supported by central pattern generators, clusters of neurons facilitating predictable rhythmic outputs (Grillner and Wallen, 1985; Grillner et al., 1995), coopted in development for speech from suckling and mastication (Lund and Kolta, 2006; Barlow et al., 2010). From comparative and evolutionary perspectives, activity of basal ganglion motor loop observed in speech activity is believed analogous to similar circuitry underlying song behavior in songbirds (Jarvis, 2004; Ackermann, 2008). Thus, while a traditional neurolinguistics framework may consider Broca’s and Wernicke’s areas as brain regions central to speech, over the last few decades, a new model of speech neurological control has emerged, emphasizing the role of BG in particular (Lieberman, 2000, 2012; Murdoch, 2001, 2009; Wildgruber et al., 2001; Ma and Suga, 2003; Radanovic and Scaff, 2003; Dronkers et al., 2007; Enard, 2011; Reimers-Kipping et al., 2011; Archakov et al., 2020; Chien et al., 2020; an extensive summary of research on the neural control of speech has been presented by Guenther, 2016).

### Structure of the basal ganglia and dopaminergic pathways

Neural substrates of motor learning, and the mesencephalic DA system that underlies it, are highly conserved across the animal kingdom (Smeets et al., 2000; Person et al., 2008; Grillner and Robertson, 2016). While differing significantly in terms of anatomical structures^4^ there is widespread continuity in the brains of songbirds and mammals as relating to organization at the level of circuitry (Reiner et al., 2004), including the BG and associated dopaminergic circuitry (Person et al., 2008; Goldberg et al., 2010), allowing for cross-species comparisons (Doupe et al., 2005; Gale and Perkel, 2010; Fee and Goldberg, 2011; Wood, 2021). Grillner and Robertson (2016, 1095) point out that in primates, “the size of the basal ganglia has expanded to a very large structure […] with the striatum being subdivided in several compartments linked to the control of different patterns of behavior.” The authors explain the expansion of the BG as having taken place in parallel with the more general expansion in complexity by the primate behavioral repertoire. In humans, the dorsal striatum can be subdivided into caudate nucleus and putamen, and again into striomes, where spiny striatal projection neurons inhibit DA neuron activity (part of the basal ganglion value-based decision-making circuitry); and matrisomes, participating in movement control (Gerfen, 1992; Stephenson-Jones et al., 2013). The division between striosomes and matrisomes is found in both humans and birds (Holt et al., 1997; Garcia-Calero et al., 2013), again suggesting an ancient evolutionary adaptation, and crucial function of the BG.

The BG is implicated in a range of behaviors, including selection of behavior, motor learning, and control of DA neuron activity and value-based decisions (Wise, 2004). The varied function of DA neurons (reviewed in Alm, 2021; see also Wood, 2021) includes the encoding of subjective goals, the initiation and preparation of movement, and instantiation of memory traces, including motor learning. In the midbrain, two nuclei—the substantia nigra pars compacta and ventral tegmental area (VTA)—are the primary producers of DA. A pathway from the VTA projects DA to the sensorimotor cortex, supplementary motor area, and dorsal premotor cortex—likely crucial for motor learning in the motor cortex (Molina-Luna et al., 2009). The primary nucleus of dopaminergic input to the BG is the striatum (Tepper et al., 2007), which also receives input from the cerebral cortex and projects to frontal lobe and brain stem nuclei (Coddington and Dudman, 2019; Klaus et al., 2019). Striatal DA release has been observed in both implicit and explicit motor performance and memory (Badgaiyan et al., 2008). Such DA neuron control is phasic, with increased activity in the presence of rewards (and decreased activity when an expected reward fails to be delivered; Howe et al., 2013), or when initiating locomotor activity (Jin and Costa, 2015). Brainstem-mediated plasticity also appears to be subject to cultural influence, with native speakers of Mandarin—a tonal language—exhibiting greater frequency-following ensemble responses to pitch contours of lexical tones, compared with native English speakers (Krishnan et al., 2005; see also Wong et al., 2009).

Fee and Goldberg (2011) proposed a common reinforcement learning mechanism underlying motor sequence learning in mammals and song learning in songbirds, based on a reward prediction biasing procedure, encompassing a BG-thalamocortical loop. Related BG circuits also contribute to the generation of variability in vocal exploration, necessary for normal mapping of song (Leblois et al., 2010). In juvenile songbirds, lesions to deep cerebellar nuclei impede song learning, with more substantial lesions resulting in greater worsening of tutor imitation (Pidoux et al., 2018). Crucially, increased DA neuron activity also facilitates long-term potentiation, the increase in synaptic strength following recent activity, including in the cerebral cortex, and including motor movement (Bailey et al., 2000; Malenka and Bear, 2004; Wise, 2004; Hosp and Luft, 2013). In addition, recent work in neurogenetics indicates that DA-genotypic individual differences are determinant of linguistic development (“the dopamine hypothesis”; Wong et al., 2012). Namely, earlier-in-life bilingual proficiency is modulated by subcortical dopamine (while later-in-life proficiency is modulated by cortical dopamine; Vaughn et al., 2016; Vaughn and Hernandez, 2018). Overall, then, basal ganglion involvement in speech, and the observed role of DA in the innervation of speech-relevant neural architectures further suggests that DA may also help guide the acquisition of speech (see also Alm, 2021).

Finally, recent work by Archakov et al. (2020) provides an important evolutionary complement. In their study, macaque monkeys were trained to produce sound sequences *via* physical manipulation of a specially designed “monkey piano.” In subsequent fMRI scans, the author observed cortical motor area activation when hearing learned melodies; simultaneous activity was also observed in the putamen of the BG (see Rauschecker, 2012, 2018). Genetics analyses of the “humanized” Forkhead Box B2 also indicate substantive involvement of the gene in the development of BG-cortical networks involved in speech (as well as language more broadly; Enard, 2011; Reimers-Kipping et al., 2011), suggesting that mutations on the gene unique to the Homo genus, contributed for the evolution of speech in ancestral hominids, as well as its proper development in modern humans (Nudel and Newbury, 2013).

### Speech and dopamine: Some clinical observations

The role of DA in speech has typically been studied in clinical contexts; namely, speech pathologies and deficits exhibit comorbidity with conditions characterized by dopaminergic dysregulation. Evidence to this effect is available from both animal models—where DA-depleted laboratory rats (*Rattus norvegicus domestica*) present with decreased call bandwidth, and maximum frequency and intensity (Ciucci et al., 2009)—and clinical research on humans, typically patients diagnosed with Parkinson’s disease (PD) or stuttering. PD is characterized by gradual brain cell death and low or falling levels of DA. Accordingly, most PD patients present with some speech pathology, most commonly hypophonic and/or monotonous speech, resulting in an articulatory undershoot (see, e.g., Ho et al., 1998). In marked contrast, stuttering—the involuntary repetition of words or segments of words—may sometimes be driven by elevated DA activity (the “dopamine hypothesis of stuttering”; Wu et al., 1997; Maguire et al., 2012; but see Alm, 2004, 2021 for nuanced accounts). The depletion of DA, characteristic of PD, degrades the local operations of the BG (Jellinger, 1990), and speech motor control is subsequently degraded also (Lieberman et al., 1992). For example, in a relevant case study, Pickett et al. (1998) observed degraded articulatory gesture sequencing in a Parkinsonian patient.

Finally, bearing on medical conditions such as PD that typically involve pathological speech, the cognitive mapping of speech-centric motor constellations remains intact; but a speaker’s ability to navigate them is disordered due to dopaminergic dysregulation, the underlying circuitry of which would otherwise maintain its reach-and-grasp-like function. Thus, while much remains unknown concerning its role in governing speech abilities, current research does indicate a role for DA in the maintenance of speech capacities across the lifespan. Less yet is known about the role of DA in phonological production learning. Nevertheless, evidence from comparative animal studies and results from simulation now suggest that dopaminergic circuitry plays a critical role in the ontogenetic development of speech motor behaviors (Gale et al., 2008; Chen and Goldberg, 2020; Kearney, 2020).

### From motor chunks to speech constellations

Neurologically, motor learning is facilitated by activity in the BG, parsing successful from unsuccessful motor behavior through comparisons with desired outcomes (Graybiel, 2005); and the cerebellum, continually adjusting fine-motor behavior (Paulin, 1993; Doya, 2000). Neurotransmission of DA significantly affects the encoding and strength of encoding of memory traces (Williams and Goldman-Rakic, 1995; Wise, 2004). In the broader context of motor learning, DA is known to contribute toward a range of behaviors. DA is crucial for enforcing associations between stimulus and subsequent rewards (Wise, 2004), and reward prediction error are, accordingly, believed to be coordinated by the BG (Wickens et al., 2003; Schultz, 2013; Gadagkar et al., 2016). Molina-Luna et al. (2009) found that lesioning dopaminergic inputs to the motor cortex in rats impaired learning of motor skills, but not execution of previously learned motor skills. Further, Gardner et al. (2018) have argued that DA be conceptualized as signaling error in both sensory and reward prediction.

Complex motor learning, underlying vocal learning, is contingent on sensory feedback (Schultz, 2007, 2013). Thus, in phonological mapping, the BG, through being part of the neural dopaminergic circuitry, likely provides the necessary emphasis for mapping speech sounds, once achieved, to its corresponding place in orosensory space, facilitating repetition across continuous interaction (Gale et al., 2008; Hoffmann et al., 2016). Simonyan et al. (2012) have previously suggested that the laryngeal motor cortex may be modulated by DA *via* its being part of the vocal BG circuitry. Neurologically, internally guided vocal explorative behavior and imitation are likely indeed enabled by common VTA-BG circuitry (Hisey et al., 2018) and guided *via* cortical-basal ganglion circuitry (Warren et al., 2011; Ali et al., 2013).

Work by Hoffmann et al. (2016) on vocal learning in Bengalese finches have demonstrated how dopaminergic inputs to the BG, such that lesions on Area X result in deficits in subjects’ vocal learning when auditory stimuli were accompanied by white noise. For explorative vocalization behavior, aspects of production corresponding to measurable acoustic outcomes (e.g., pitch, amplitude) may be controlled by separate neuronal ensembles (Sober et al., 2008). Based on their observations, Hoffmann et al. (2016) argued that vocal plasticity is selectively reinforced *via* dopaminergic inputs to the BG (Hoffmann et al., 2016, p. 2176), mirroring an equivalent process in perception learning (Gale and Perkel, 2010). Similarly, in humans, imitation is also presumed to guide children’s acquisition of speech (Messum, 2008). Production itself is likely regulated *via* inputs from the cerebellum (Ackermann, 2008), as indicated by work on the song production pathways of zebra finches by Pidoux et al. (2018).

The cerebral DA network thus appears to provide a mechanism for the automatization of motor movement sequence “chunks”—that is, sequences composed from otherwise isolated movements—to be coordinated and executed in tandem, or in sequence (Marsden and Obeso, 1994; Alm, 2021). Basal ganglion–cerebellar dopaminergic circuitry thus provides the necessary emphasis for mapping a song component or fragment, once achieved, to its corresponding motor activity constellation in syringeal–orosensory space, enabling replicated matching over repeated vocalizations across time (see Gale et al., 2008).^5^ Thus, it is here supposed that generalized mechanisms have evolved convergently for the mapping of constellations of motor activity in domains of mouth and larynx (in mammals) or syrinx (in songbirds), to the bounded auditory outputs to which their innervation corresponds.

## Motor constellation theory

**Figure 2:**
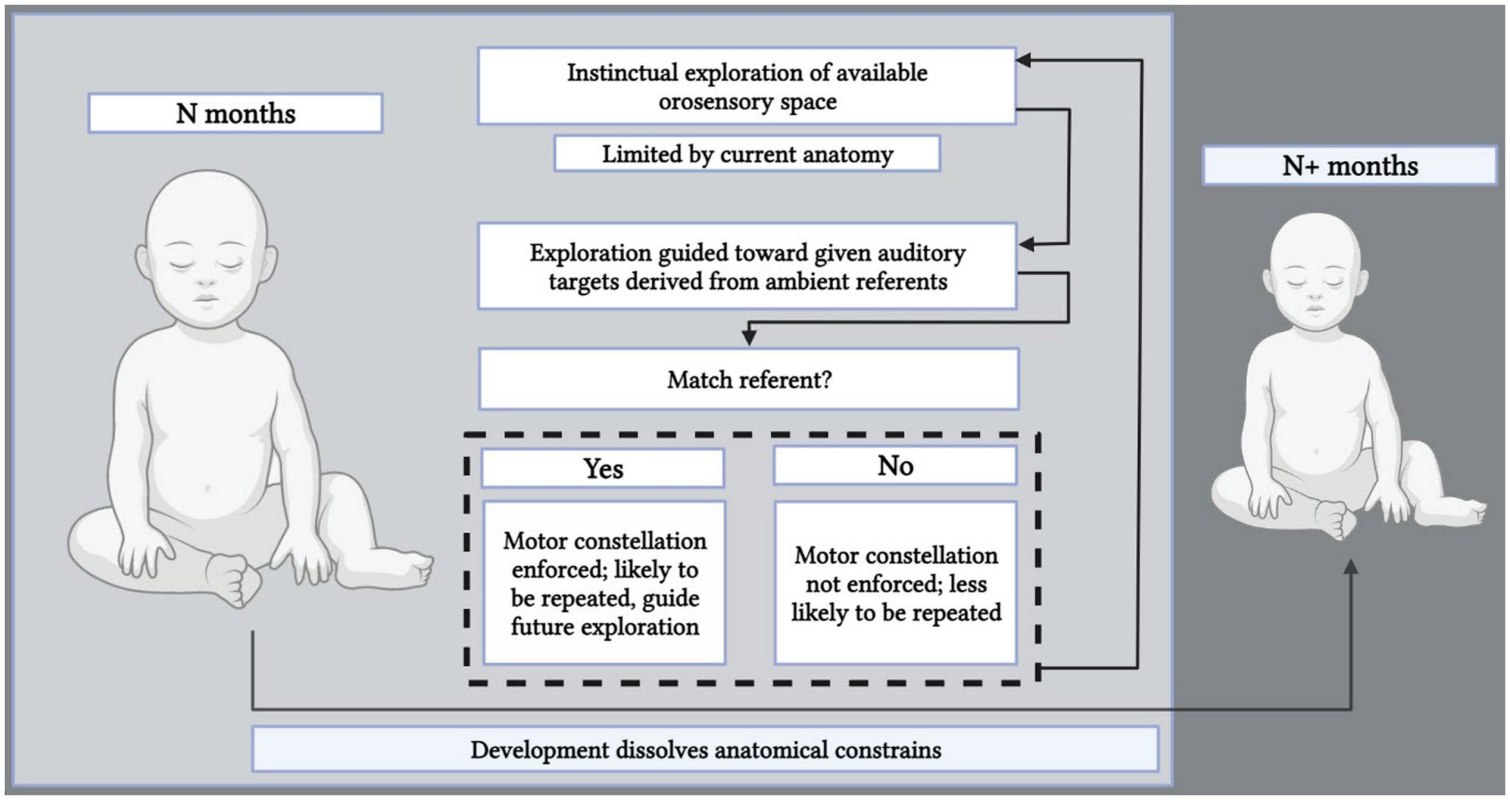
Motor constellation theory: A sketch of the proposed model.

The purpose of the present text was to indicate the biological underpinnings of infants’ phonological mapping. To this goal, the motor constellation theory of phonological development (MC) was presented. The theory posits that human infants are born with the instinct to explore orosensory space through tactile sensory motor behavioral and auditory feedback. Babbling is the result of successful such exploration, giving rise to emergent pseudo-segmental phonetic properties. Continuous perceptual-motor mapping facilitates the acquisition of language-specific phonemic repertoires, and gives rise to phonemes proper, defined as discrete target positions in cognitive–orosensory space. Babble is thus gradually replaced by elective values in sound space, selected *via* interaction with ingroup members, enforced and reinforced *via* cerebellar–basal ganglion circuitry for dopaminergic signaling, which instantiates encoding of combinations of motor sensory and auditory perceptual features, and providing the necessary mechanism by which speech sounds are mapped onto corresponding laryngeal–orosensory motor activity constellations. Once achieved, any reinforced combinatory pattern becomes more easily repeatable through continuous reinstatement (see Figure 2). Continuous and ritualized reuse of a given constellation of motor coordinates leads to the formation and memorization of phonetic concepts in memory; motor constellations thus become the roadmaps by which a phonetic concept is explored, learned, mapped, and maintained across time in the individual speaker.

### Some considerations for modeling

The dopaminergic innervation of speech behavior thus proposed, we next seek to model—and ultimately to simulate—phonological production development. Vocal learning is (at least in part) intrinsically motivated, as is evident from both anthropological evidence that infants learn to speak normally even in cultures where they are rarely if ever addressed directly (Ochs and Schieffelin, 2009); observations of songbirds’ song learning (Marler, 1970); and simulation and modeling approaches (e.g., Chen and Goldberg, 2020). In his work on birdsong, Marler (1970, 670) speculated that “the process of vocal imitation may prove to be essentially self-reinforcing in the cases both of juvenile birds and infant humans and thus basically be independent of reward by the parent.”

Researchers investigating song learning have also previously hypothesized the importance of motor exploration. It was first noted by Metfessel (1935) that domestic canaries (*Serinus canaria domestica*) learn to sing through a process of improvisation, and that this process still occurs even in the absence of external referent sources. Later work showed how the same species can also learn by imitation (Poulsen, 1959; Marler and Waser, 1977; see also Nottebohm et al., 1986). Even in adulthood (some) songbirds are capable of adaptive fundamental frequency shift in vocalization, shifting the fundamental frequency of some targeted portion of a song to avoid disruption, consistent with some degree of flexibility across the lifespan (Tumer and Brainard, 2007). While DA has traditionally been studied in the context of reinforcement learning—trial-and-error based environmental sampling with the goal of attaining maximum value (see Wood, 2021), complex motor behaviors such as song— and therefore, possibly also speech—likely involve the utilization of multiple simultaneous learning strategies and mechanisms (Guenther, 2016; Krakauer et al., 2019; Wood, 2021).

Human infants’ imitative vocalizations are seemingly guided by memorized phonological patterns (Fry, 1966; Kuhl and Meltzoff, 1996), and phonological production learning likely represents such a case of simultaneous model-based and model-free reinforcement learning, where prior motor-sound equivalence experience helps guide increasingly sophisticated attempts at phonological matching of own-speech output, with that observed prior; that is, learning by reference sensory-prediction error. Constellations thus enforced become more easily reachable across future interactions *via* Hebbian learning, the strengthening of synaptic connection *via* repeated signaling activity (Hebb, 1949; Marsden and Obeso, 1994; Gale et al., 2008; Hoffmann et al., 2016; Wood, 2021). Indeed, even in adults, greater white matter content predicts faster phonetic learning (Golestani et al., 2002). Because of concerns both ethical and methodological, however, the hypothesis here presented is not available to direct investigation. Modern neuroscientific tools are not yet sophisticated enough to track dopaminergic flow non-invasively, a problem multiplied when subjects are non-verbal and unable to consent to experiment procedures.

Implications discussed, do however, open up new avenues for computational and simulation modeling (Lindblom, 2000; Guenther and Vladusich, 2012). In particular, one promising novel avenue for future modeling work is that of actor-critic methods, where an actor is synonymous with policy—the appropriate action given a certain state—and critic corresponds to a value function—the estimated return from committing to a policy (see Konda and Tsitsiklis, 2003). Chen and Goldberg (2020) have recently presented an actor-critic reinforcement model of song learning in songbirds. The authors suggest that both note correctness and quality, unexpectedly achieved in improvised vocalization, trigger DA neuron activation. Additionally, Kearney (2020) has also presented results of actor-critic simulations of song learning, showing that (1) disruption of midbrain DA circuit input (“actors”) at the moment of auditory feedback, impairs learning, as does and (2) disruption of downstream premotor region activity at early preparatory stages of vocalization (see also Gale et al., 2008; Gale and Perkel, 2010). To the knowledge of the author, no actor-critic model yet presented has attempted to simulate infants’ phonological development. Nevertheless, these promising early results merit further exploration, and application to vocal learning in human infants also.

### Some considerations for clinical practice

Motor constellation also has important implications for understanding early-in-life speech pathologies, such as stuttering. DA functioning is indeed highly implicated in stuttering behavior (Wu et al., 1997; Alm, 2004; Maguire et al., 2012). While the exact nature of the relationship is not certain, results of various interventions have pointed to lessened stuttering following treatment with DA agonists (e.g., Levodopa; Anderson et al., 1999) and worsened stuttering following treatment with DA antagonists, often interpreted as evidence that an excess DA drives stuttering (e.g., Rosenberger et al., 1976; for an overview, see Maguire et al., 2020; but see also Alm, 2021). The relationship is further complicated by a variety of individual variables. For example, genotypical makeup likely plays a determinant role in the development of the condition, as is evident from twin studies (Yairi and Ambrose, 2013) and genetics research (Montag et al., 2012). However, while children identified as carrying genotypic traits associated with greater levels of DA exhibit higher levels of linguistic proficiencies (Wong et al., 2012; Vaughn and Hernandez, 2018), it is as yet not known whether children exhibiting stuttering (or other speech disorders) can be similarly characterized (though results of twin studies point to this being so). Future work should aim to address this issue.

Finally, Ashby and colleagues (Ashby et al., 2010; Hélie et al., 2015) have proposed that BG serve to ritualize motor sequences, such that once learned they can be executed without direct BG involvement (BG may still be central to execution during early developmental periods; the “Ashby model”). That is, the role of DA in speech mapping and maintenance is likely inconsistent, changing significantly across the lifespan, with DA release in the BG affecting vigor (but not motor sequence initiation) later in life. Stuttering disfluencies also vary significantly with situational variables, with more demanding speech situations causing greater stuttering (Craig, 1990; Perkins et al., 1991; Alm, 2014), again suggesting an effect of higher cognition. As a framework of phonological development, MC is consistent with these views. Assuming DA-innervated reuse of motor constellations in early life, childhood stuttering may result from dysregulated DA innervation of ritualized constellations.

### Concluding comments

Motor constellation sidesteps common theoretical misgivings in the construction of theories of language acquisition postulated *post hoc* based on observed data (Chapman, 2000; Lindblom, 2000). It presents researchers with an account of phonological development that (1) assimilates observations of human early speech acquisition and (2) is rooted in principles of the natural sciences and neuroscience underlying motor learning, and (3) affords integration with phonetic, neuropsychological, and evolutionary sciences. Finally, while empirical testing in human infants—due to technological limitations of contemporary brain imaging techniques, as well as ethical considerations—may not be feasible, MC affords both computational modeling and simulation approaches, and has additional implications for clinical work. It is the hope of the author that the present text helps guide such efforts in the future.

## Data availability statement

The original contributions presented in the study are included in the article/supplementary material, further inquiries can be directed to the corresponding author.

## Author contributions

The author confirms being the sole contributor of this work and has approved it for publication.

## Conflict of interest

The author declares that the research was conducted in the absence of any commercial or financial relationships that could be construed as a potential conflict of interest.

## Publisher’s note

All claims expressed in this article are solely those of the authors and do not necessarily represent those of their affiliated organizations, or those of the publisher, the editors and the reviewers. Any product that may be evaluated in this article, or claim that may be made by its manufacturer, is not guaranteed or endorsed by the publisher.

## Footnotes

^1^Note that as tonal elements are delineated by changes in *f*_0_, the trajectory of tone acquisition outlined by Wong and Strange (2017) involves laryngeal, as opposed to supralaryngeal development.

^2^In this context it is worth noting that the degree to which the organization of the songbird brain parallels that of humans (and other mammals) is subject to extensive, as of yet unsettled debate (Reiner et al., 2004; Petkov and Jarvis, 2012; Olkowicz et al., 2016; Prather et al., 2017).

^3^For language learning (as well as phonological learning), "Procedural/Declarative" model of Ullman (2001) similarly argues for a role of BG in ordering mental grammar.

^4^Aves lack the mammalian prefrontal cortex, but seemingly possess a functionally comparable structure in the nidopallium caudolaterale (see Güntürkün, 2005).

^5^It is not here suggested, then, that songbirds’ mapping of song fragments is in any way equivalent to human language grammar (though such arguments have been made elsewhere; e.g., Abe and Watanabe, 2011).

## References

[ref1] Abe K. Watanabe D. (2011). Songbirds possess the spontaneous ability to discriminate syntactic rules. Nat. Neurosci. 14, 1067–1074. doi: 10.1038/nn.2869, PMID: 21706017

[ref2] Ackermann H. (2008). Cerebellar contributions to speech production and speech perception: psycholinguistic and neurobiological perspectives. Trends Neurosci. 31, 265–272. doi: 10.1016/j.tins.2008.02.011, PMID: 18471906

[ref3] Ackermann H. Ziegler W. (2010). Brain mechanisms underlying speech motor control. Handbook Phonet. Sci. 2, 202–250. doi: 10.1002/9781444317251.ch6

[ref4] Alexander M. P. Naeser M. A. Palumbo C. L. (1987). Correlations of subcortical lesion sites and aphasia profiles. Brain 110, 961–988. doi: 10.1093/brain/110.4.961, PMID: 3651803

[ref5] Ali F. Otchy T. M. Pehlevan C. Fantana A. L. Burak Y. Ölveczky B. P. (2013). The basal ganglia is necessary for learning spectral, but not temporal, features of birdsong. Neuron 80, 494–506. doi: 10.1016/j.neuron.2013.07.049, PMID: 24075977 PMC3929499

[ref6] Alm P. A. (2004). Stuttering and the basal ganglia circuits: a critical review of possible relations. J. Commun. Disord. 37, 325–369. doi: 10.1016/j.jcomdis.2004.03.001, PMID: 15159193

[ref7] Alm P. A. (2014). Stuttering in relation to anxiety, temperament, and personality: review and analysis with focus on causality. J. Fluen. Disord. 40, 5–21. doi: 10.1016/j.jfludis.2014.01.004, PMID: 24929463

[ref8] Alm P. A. (2021). The dopamine system and Automatization of movement sequences: a review with relevance for speech and stuttering. Front. Hum. Neurosci. 15:661880. doi: 10.3389/fnhum.2021.661880, PMID: 34924974 PMC8675130

[ref9] Anderson J. M. Hughes J. D. Rothi L. J. G. Crucian G. P. Heilman K. M. (1999). Developmental stuttering and Parkinson’s disease: the effects of levodopa treatment. J. Neurol. Neurosurg. Psychiatry 66, 776–778. doi: 10.1136/jnnp.66.6.776, PMID: 10329754 PMC1736378

[ref10] Archakov D. DeWitt I. Kuśmierek P. Ortiz-Rios M. Cameron D. Cui D.. (2020). Auditory representation of learned sound sequences in motor regions of the macaque brain. Proc. Natl. Acad. Sci. 117, 15242–15252. doi: 10.1073/pnas.1915610117, PMID: 32541016 PMC7334521

[ref11] Armstrong D. M. Marple-Horvat D. E. (1996). Role of the cerebellum and motor cortex in the regulation of visually controlled locomotion. Can. J. Physiol. Pharmacol. 74, 443–455. doi: 10.1139/y96-044, PMID: 8828890

[ref12] Ashby F. G. Turner B. O. Horvitz J. C. (2010). Cortical and basal ganglia contributions to habit learning and automaticity. Trends Cogn. Sci. 14, 208–215. doi: 10.1016/j.tics.2010.02.001, PMID: 20207189 PMC2862890

[ref13] Badgaiyan R. D. Fischman A. J. Alpert N. M. (2008). Explicit motor memory activates the striatal dopamine system. Neuroreport 19, 409–412. doi: 10.1097/WNR.0b013e3282f6435f, PMID: 18287937

[ref14] Bailey C. H. Giustetto M. Huang Y. Y. Hawkins R. D. Kandel E. R. (2000). Is heterosynaptic modulation essential for stabilizing Hebbian plasticity and memory. Nat. Rev. Neurosci. 1, 11–20. doi: 10.1038/35036191, PMID: 11252764

[ref15] Baptista L. F. Schuchmann K. L. (1990). Song learning in the Anna hummingbird (Calypte anna). Ethology 84, 15–26. doi: 10.1111/j.1439-0310.1990.tb00781.x

[ref16] Barlow S. M. Radder J. P. L. Radder M. E. Radder A. K. (2010). Central pattern generators for orofacial movements and speech. Handbook Behav. Neurosci. 19, 351–369. doi: 10.1016/B978-0-12-374593-4.00033-4

[ref17] Barr H. J. Wall E. M. Woolley S. C. (2021). Dopamine in the songbird auditory cortex shapes auditory preference. Curr. Biol. 31, 4547–4559.e5. doi: 10.1016/j.cub.2021.08.005, PMID: 34450091

[ref18] Bass A. H. Gilland E. H. Baker R. (2008). Evolutionary origins for social vocalization in a vertebrate hindbrain–spinal compartment. Science 321, 417–421. doi: 10.1126/science.1157632, PMID: 18635807 PMC2582147

[ref20] Bolhuis J. J. (1991). Mechanisms of avian imprinting: a review. Biol. Rev. 66, 303–345. doi: 10.1111/j.1469-185X.1991.tb01145.x 1801945

[ref21] Bolhuis J. J. Gahr M. (2006). Neural mechanisms of birdsong memory. Nat. Rev. Neurosci. 7, 347–357. doi: 10.1038/nrn1904, PMID: 16760915

[ref22] Bolhuis J. J. Moorman S. (2015). Birdsong memory and the brain: in search of the template. Neurosci. Biobehav. Rev. 50, 41–55. doi: 10.1016/j.neubiorev.2014.11.019, PMID: 25459663

[ref23] Bolhuis J. J. Okanoya K. Scharff C. (2010). Twitter evolution: converging mechanisms in birdsong and human speech. Nat. Rev. Neurosci. 11, 747–759. doi: 10.1038/nrn2931, PMID: 20959859

[ref24] Bouchard K. E. Mesgarani N. Johnson K. Chang E. F. (2013). Functional organization of human sensorimotor cortex for speech articulation. Nature 495, 327–332. doi: 10.1038/nature11911, PMID: 23426266 PMC3606666

[ref50] Boysson-Bardies B. de. Vihman M. M. Roug-Hellichius L. Durand C. Landberg I. Arao F. (1992). “Material evidence of infant selection from the target language: a cross-linguistic phonetic study,” in Phonological Development: Models, Research, Implications. eds. Ferguson C. A. Menn L. Stoel-Gammon C. (York, Timonium, MD), 369–391.

[ref25] Bradbury J. W. Balsby T. J. (2016). The functions of vocal learning in parrots. Behav. Ecol. Sociobiol. 70, 293–312. doi: 10.1007/s00265-016-2068-4

[ref26] Brainard M. S. Doupe A. J. (2000). Auditory feedback in learning and maintenance of vocal behavior. Nat. Rev. Neurosci. 1, 31–40. doi: 10.1038/35036205 11252766

[ref27] Broca P. (1861). Remarks on the seat of the faculty of articulated language, following an observation of aphemia (loss of speech). Bull. Soc. Anat. 6, 330–357.

[ref28] Browman C. P. Goldstein L. (1989). Articulatory gestures as phonological units. Phonology 6, 201–251. doi: 10.1017/S0952675700001019

[ref29] Brown S. Ngan E. Liotti M. (2008). A larynx area in the human motor cortex. Cereb. Cortex 18, 837–845. doi: 10.1093/cercor/bhm131 17652461

[ref30] Brown S. Yuan Y. Belyk M. (2021). Evolution of the speech-ready brain: the voice/jaw connection in the human motor cortex. J. Comp. Neurol. 529, 1018–1028. doi: 10.1002/cne.24997, PMID: 32720701

[ref31] Burnett T. A. Freedland M. B. Larson C. R. Hain T. C. (1998). Voice F0 responses to manipulations in pitch feedback. J. Acoust. Soc. Am. 103, 3153–3161. doi: 10.1121/1.423073, PMID: 9637026

[ref33] Chapman R. S. (2000). Children’s language learning: an interactionist perspective. J. Child Psychol. Psychiatry Allied Discip. 41, 33–54. doi: 10.1017/S0021963099004953, PMID: 10763675

[ref34] Chen R. Goldberg J. H. (2020). Actor-critic reinforcement learning in the songbird. Curr. Opin. Neurobiol. 65, 1–9. doi: 10.1016/j.conb.2020.08.005, PMID: 32898752 PMC7769887

[ref35] Chen L. M. Kent R. D. (2010). Segmental production in mandarin-learning infants. J. Child Lang. 37, 341–371. doi: 10.1017/S0305000909009581, PMID: 19490748

[ref36] Cheour M. Ceponiene R. Lehtokoski A. Luuk A. Allik J. Alho K.. (1998). Development of language-specific phoneme representations in the infant brain. Nat. Neurosci. 1, 351–353. doi: 10.1038/1561, PMID: 10196522

[ref37] Chien P. J. Friederici A. D. Hartwigsen G. Sammler D. (2020). Neural correlates of intonation and lexical tone in tonal and non-tonal language speakers. Hum. Brain Mapp. 41, 1842–1858. doi: 10.1002/hbm.24916, PMID: 31957928 PMC7268089

[ref38] Chomsky N. (1986). Knowledge of Language: Its Nature, Origin, and Use. New York: Praeger Greenwood Publishing Group.

[ref39] Chomsky N. (2002). Syntactic Structures (2nd Edn.). Berlin, NY: Mouton de Gruyter

[ref40] Chrabaszcz A. Neumann W. J. Stretcu O. Lipski W. J. Bush A. Dastolfo-Hromack C. A.. (2019). Subthalamic nucleus and sensorimotor cortex activity during speech production. J. Neurosci. 39, 2698–2708. doi: 10.1523/JNEUROSCI.2842-18.2019, PMID: 30700532 PMC6445998

[ref41] Ciucci M. R. Ahrens A. M. Ma S. T. Kane J. R. Windham E. B. Woodlee M. T.. (2009). Reduction of dopamine synaptic activity: degradation of 50-kHz ultrasonic vocalization in rats. Behav. Neurosci. 123, 328–336. doi: 10.1037/a0014593, PMID: 19331456 PMC2737695

[ref001] Coddington L. T. Dudman J. T. (2019). Learning from action: reconsidering movement signaling in midbrain dopamine neuron activity. Neuron 104, 63–77. doi: 10.1016/j.neuron.2019.08.036 31600516

[ref42] Colquitt B. M. Merullo D. P. Konopka G. Roberts T. F. Brainard M. S. (2021). Cellular transcriptomics reveals evolutionary identities of songbird vocal circuits. Science 371:eabd9704. doi: 10.1126/science.abd9704, PMID: 33574185 PMC8136249

[ref43] Craig A. (1990). An investigation into the relationship between anxiety and stuttering. J. Speech Hear. Disord. 55, 290–294. doi: 10.1044/jshd.5502.290, PMID: 2329791

[ref44] Cummings J. L. (1993). Frontal-subcortical circuits and human behavior. Arch. Neurol. 50, 873–880. doi: 10.1001/archneur.1993.00540080076020 8352676

[ref45] Daou A. Margoliash D. (2021). Intrinsic plasticity and birdsong learning. Neurobiol. Learn. Mem. 180:107407. doi: 10.1016/j.nlm.2021.107407, PMID: 33631346 PMC8076075

[ref46] de Boysson-Bardies B. (2001). How Language Comes to Children: From Birth to Two Years MIT Press.

[ref47] de Boysson-Bardies B. Hallé P. Sagart L. Durand C. (1989). A crosslinguistic investigation of vowel formants in babbling. J. Child Lang. 16, 1–17. doi: 10.1017/S0305000900013404, PMID: 2925806

[ref48] De Boysson-Bardies B. Sagart L. Bacri N. (1981). Phonetic analysis of late babbling: a case study of a French child. J. Child Lang. 8, 511–524. doi: 10.1017/S0305000900003408, PMID: 7309814

[ref49] de Boysson-Bardies B. Sagart L. Durand C. (1984). Discernible differences in the babbling of infants according to target language. J. Child Lang. 11, 1–15. doi: 10.1017/S0305000900005559, PMID: 6699104

[ref51] de Villers-Sidani E. Merzenich M. M. (2011). Lifelong plasticity in the rat auditory cortex: basic mechanisms and role of sensory experience. Prog. Brain Res. 191, 119–131. doi: 10.1016/B978-0-444-53752-2.00009-6, PMID: 21741548

[ref52] Denes P. B. Pinson E. (1963). The Speech Chain: The Physics and Biology of Spoken Language, Macmillan.

[ref53] Di Pellegrino G. Fadiga L. Fogassi L. Gallese V. Rizzolatti G. (1992). Understanding motor events: a neurophysiological study. Exp. Brain Res. 91, 176–180. doi: 10.1007/BF00230027, PMID: 1301372

[ref54] Dichter B. K. Breshears J. D. Leonard M. K. Chang E. F. (2018). The control of vocal pitch in human laryngeal motor cortex. Cells 174, 21–31.e9. doi: 10.1016/j.cell.2018.05.016, PMID: 29958109 PMC6084806

[ref55] Doupe A. J. Kuhl P. K. (1999). Birdsong and human speech: common themes and mechanisms. Annu. Rev. Neurosci. 22, 567–631. doi: 10.1146/annurev.neuro.22.1.567, PMID: 10202549

[ref56] Doupe A. J. Perkel D. J. Reiner A. Stern E. A. (2005). Birdbrains could teach basal ganglia research a new song. Trends Neurosci. 28, 353–363. doi: 10.1016/j.tins.2005.05.005, PMID: 15935486

[ref57] Doya K. (2000). Complementary roles of basal ganglia and cerebellum in learning and motor control. Curr. Opin. Neurobiol. 10, 732–739. doi: 10.1016/S0959-4388(00)00153-7, PMID: 11240282

[ref58] Drew T. (1993). Motor cortical activity during voluntary gait modifications in the cat. I. Cells related to the forelimbs. J. Neurophysiol. 70, 179–199. doi: 10.1152/jn.1993.70.1.179, PMID: 8360715

[ref59] Drew T. Andujar J. E. Lajoie K. Yakovenko S. (2008). Cortical mechanisms involved in visuomotor coordination during precision walking. Brain Res. Rev. 57, 199–211. doi: 10.1016/j.brainresrev.2007.07.017, PMID: 17935789

[ref60] Dronkers N. F. Plaisant O. Iba-Zizen M. T. Cabanis E. A. (2007). Paul Broca's historic cases: high resolution MR imaging of the brains of Leborgne and Lelong. Brain 130, 1432–1441. doi: 10.1093/brain/awm042, PMID: 17405763

[ref61] Egnor S. R. Hauser M. D. (2004). A paradox in the evolution of primate vocal learning. Trends Neurosci. 27, 649–654. doi: 10.1016/j.tins.2004.08.009, PMID: 15474164

[ref62] Eibl-Eibesfeldt I. (1973). “The expressive behavior of the deaf-andblind-born,” in Social Communication and Movement. eds. von Cranach M. Vine I. (San Diego, CA: Academic Press), 163–194.

[ref63] Elman J. L. (1981). Effects of frequency-shifted feedback on the pitch of vocal productions. J. Acoust. Soc. Am. 70, 45–50. doi: 10.1121/1.386580, PMID: 7264071

[ref64] Elsner B. (2007). Infants’ imitation of goal-directed actions: the role of movements and action effects. Acta Psychol. 124, 44–59. doi: 10.1016/j.actpsy.2006.09.006, PMID: 17078915

[ref65] Enard W. (2011). FOXP2 and the role of cortico-basal ganglia circuits in speech and language evolution. Curr. Opin. Neurobiol. 21, 415–424. doi: 10.1016/j.conb.2011.04.008, PMID: 21592779

[ref66] Farries M. A. (2004). The avian song system in comparative perspective. Ann. N. Y. Acad. Sci. 1016, 61–76. doi: 10.1196/annals.1298.007, PMID: 15313770

[ref67] Fee M. S. Goldberg J. H. (2011). A hypothesis for basal ganglia-dependent reinforcement learning in the songbird. Neuroscience 198, 152–170. doi: 10.1016/j.neuroscience.2011.09.069, PMID: 22015923 PMC3221789

[ref68] Fernald A. (1991). Prosody in speech to children: Prelinguistic and linguistic functions. Ann. Child Dev. 8, 43–80.

[ref69] Field T. M. Woodson R. Cohen D. Greenberg R. Garcia R. Collins K. (1983). Discrimination and imitation of facial expressions by term and preterm neonates. Infant Behav. Dev. 6, 485–489. doi: 10.1016/S0163-6383(83)90316-8

[ref70] Field T. M. Woodson R. Greenberg R. Cohen D. (1982). Discrimination and imitation of facial expression by neonates. Science 218, 179–181. doi: 10.1126/science.7123230, PMID: 7123230

[ref71] Friederici A. D. Gierhan S. M. (2013). The language network. Curr. Opin. Neurobiol. 23, 250–254. doi: 10.1016/j.conb.2012.10.002 23146876

[ref002] Fry D. B. (1966). “The development of the phonological system in the normal and the deaf child,” in The Genesis of Language: A Psycholinguistic Approach. eds. Smith F. Miller G. (Cambridge, MA: MIT Press), 187–206.

[ref72] Gadagkar V. Puzerey P. A. Chen R. Baird-Daniel E. Farhang A. R. Goldberg J. H. (2016). Dopamine neurons encode performance error in singing birds. Science 354, 1278–1282. doi: 10.1126/science.aah6837, PMID: 27940871 PMC5464363

[ref73] Gale S. D. Perkel D. J. (2010). A basal ganglia pathway drives selective auditory responses in songbird dopaminergic neurons via disinhibition. J. Neurosci. 30, 1027–1037. doi: 10.1523/JNEUROSCI.3585-09.2010, PMID: 20089911 PMC2824341

[ref74] Gale S. D. Person A. L. Perkel D. J. (2008). A novel basal ganglia pathway forms a loop linking a vocal learning circuit with its dopaminergic input. J. Comp. Neurol. 508, 824–839. doi: 10.1002/cne.21700, PMID: 18398824

[ref75] Garcia-Calero E. Bahamonde O. Martinez S. (2013). Differences in number and distribution of striatal calbindin medium spiny neurons between a vocal-learner (Melopsittacus undulatus) and a non-vocal learner bird (Colinus virginianus). Front. Neuroanat. 7:46. doi: 10.3389/fnana.2013.00046 24391552 PMC3867642

[ref003] Gardner M. P. Schoenbaum G. Gershman S. J. (2018). Rethinking dopamine as generalized prediction error. Proc. Biol. Sci. 285:20181645. doi: 10.1098/rspb.2018.1645 30464063 PMC6253385

[ref76] Gay T. Lindblom B. Lubker J. (1981). Production of bite-block vowels: acoustic equivalence by selective compensation. J. Acoust. Soc. Am. 69, 802–810. doi: 10.1121/1.385591, PMID: 7240561

[ref77] Gerfen C. R. (1992). The neostriatal mosaic: multiple levels of compartmental organization. Adv. Neurosci. Schizophrenia, 43–59. doi: 10.1007/978-3-7091-9211-5_4, PMID: 1374971

[ref78] Gibson J. J. (1979). The Ecological Approach to Visual Perception. New York: Houghton Mifflin

[ref79] Goldberg J. H. Adler A. Bergman H. Fee M. S. (2010). Singing-related neural activity distinguishes two putative pallidal cell types in the songbird basal ganglia: comparison to the primate internal and external pallidal segments. J. Neurosci. 30, 7088–7098. doi: 10.1523/JNEUROSCI.0168-10.2010, PMID: 20484651 PMC2874984

[ref80] Goldstein M. H. King A. P. West M. J. (2003). Social interaction shapes babbling: testing parallels between birdsong and speech. Proc. Natl. Acad. Sci. 100, 8030–8035. doi: 10.1073/pnas.1332441100, PMID: 12808137 PMC164707

[ref81] Goldstein M. H. Schwade J. A. (2008). Social feedback to infants' babbling facilitates rapid phonological learning. Psychol. Sci. 19, 515–523. doi: 10.1111/j.1467-9280.2008.02117.x, PMID: 18466414

[ref82] Golestani N. Paus T. Zatorre R. J. (2002). Anatomical correlates of learning novel speech sounds. Neuron 35, 997–1010. doi: 10.1016/S0896-6273(02)00862-0, PMID: 12372292

[ref83] Gracco V. L. Tremblay P. Pike B. (2005). Imaging speech production using fMRI. NeuroImage 26, 294–301. doi: 10.1016/j.neuroimage.2005.01.033 15862230

[ref84] Graybiel A. M. (2005). The basal ganglia: learning new tricks and loving it. Curr. Opin. Neurobiol. 15, 638–644. doi: 10.1016/j.conb.2005.10.006, PMID: 16271465

[ref85] Green J. R. Nip I. S. (2010). Some organization principles in early speech development. Speech Motor Control 10, 171–188. doi: 10.1093/acprof:oso/9780199235797.003.0010

[ref86] Greenwalt C. H. (1968). Bird Song: Acoustics and Physiology. Washington, D.C.: Smithsonian Institution Press

[ref87] Grillner S. Deliagina T. El Manira A. Hill R. H. Orlovsky G. N. Wallén P.. (1995). Neural networks that co-ordinate locomotion and body orientation in lamprey. Trends Neurosci. 18, 270–279. doi: 10.1016/0166-2236(95)80008-P, PMID: 7571002

[ref88] Grillner S. Robertson B. (2016). The basal ganglia over 500 million years. Curr. Biol. 26, R1088–R1100. doi: 10.1016/j.cub.2016.06.041, PMID: 27780050

[ref89] Grillner S. Wallen P. (1985). Central pattern generators for locomotion, with special reference to vertebrates. Annu. Rev. Neurosci. 8, 233–261. doi: 10.1146/annurev.ne.08.030185.001313, PMID: 2984978

[ref90] Guenther F. H. (1994). A neural network model of speech acquisition and motor equivalent speech production. Biol. Cybern. 72, 43–53. doi: 10.1007/BF00206237, PMID: 7880914

[ref91] Guenther F. H. (1995). Speech sound acquisition, coarticulation, and rate effects in a neural network model of speech production. Psychol. Rev. 102, 594–621. doi: 10.1037/0033-295X.102.3.594, PMID: 7624456

[ref92] Guenther F. H. (2006). Cortical interactions underlying the production of speech sounds. J. Commun. Disord. 39, 350–365. doi: 10.1016/j.jcomdis.2006.06.013, PMID: 16887139

[ref93] Guenther F. H. (2016). Neural Control of Speech MIT Press. *Vol 15*.

[ref94] Guenther F. H. Vladusich T. (2012). A neural theory of speech acquisition and production. J. Neurolinguistics 25, 408–422. doi: 10.1016/j.jneuroling.2009.08.006, PMID: 22711978 PMC3375605

[ref95] Güntürkün O. (2005). The avian ‘prefrontal cortex’ and cognition. Curr. Opin. Neurobiol. 15, 686–693. doi: 10.1016/j.conb.2005.10.003, PMID: 16263260

[ref96] Hage S. R. Nieder A. (2016). Dual neural network model for the evolution of speech and language. Trends Neurosci. 39, 813–829. doi: 10.1016/j.tins.2016.10.006, PMID: 27884462

[ref97] Hahnloser R. H. Kotowicz A. (2010). Auditory representations and memory in birdsong learning. Curr. Opin. Neurobiol. 20, 332–339. doi: 10.1016/j.conb.2010.02.011, PMID: 20307967

[ref98] Hammer M. J. Krueger M. A. (2014). Voice-related modulation of mechanosensory detection thresholds in the human larynx. Exp. Brain Res. 232, 13–20. doi: 10.1007/s00221-013-3703-1, PMID: 24217976 PMC3979554

[ref99] Harrison D. F. N. (1995). The Anatomy and Physiology of the Mammalian Larynx Cambridge University Press.

[ref100] Hebb D. (1949). The Organization of Behavior: A Neuropsychological Theory. New York: John Wiley & Sons

[ref101] Hélie S. Ell S. W. Ashby F. G. (2015). Learning robust cortico-cortical associations with the basal ganglia: an integrative review. Cortex 64, 123–135. doi: 10.1016/j.cortex.2014.10.011, PMID: 25461713

[ref102] Hisey E. Kearney M. G. Mooney R. (2018). A common neural circuit mechanism for internally guided and externally reinforced forms of motor learning. Nat. Neurosci. 21, 589–597. doi: 10.1038/s41593-018-0092-6, PMID: 29483664 PMC5963939

[ref103] Ho A. K. Iansek R. Marigliani C. Bradshaw J. L. Gates S. (1998). Speech impairment in a large sample of patients with Parkinson's disease. Behav. Neurol. 11, 131–137. doi: 10.1155/1999/327643, PMID: 22387592

[ref104] Hoffmann L. A. Saravanan V. Wood A. N. He L. Sober S. J. (2016). Dopaminergic contributions to vocal learning. J. Neurosci. 36, 2176–2189. doi: 10.1523/JNEUROSCI.3883-15.2016, PMID: 26888928 PMC4756153

[ref105] Holt D. J. Graybiel A. M. Saper C. B. (1997). Neurochemical architecture of the human striatum. J. Comp. Neurol. 384, 1–25. doi: 10.1002/(SICI)1096-9861(19970721)384:1<1::AID-CNE1>3.0.CO;2-5, PMID: 9214537

[ref106] Hosp J. A. Luft A. R. (2013). Dopaminergic meso-cortical projections to M1: role in motor learning and motor cortex plasticity. Front. Neurol. 4:145. doi: 10.3389/fneur.2013.00145 24109472 PMC3791680

[ref107] Houde J. F. Jordan M. I. (1998). Sensorimotor adaptation in speech production. Science 279, 1213–1216. doi: 10.1126/science.279.5354.1213 9469813

[ref108] Howe M. W. Tierney P. L. Sandberg S. G. Phillips P. E. Graybiel A. M. (2013). Prolonged dopamine signalling in striatum signals proximity and value of distant rewards. Nature 500, 575–579. doi: 10.1038/nature12475, PMID: 23913271 PMC3927840

[ref109] Hsu H. C. Fogel A. Cooper R. B. (2000). Infant vocal development during the first 6 months: speech quality and melodic complexity. Infant Child Dev. 9, 1–16. doi: 10.1002/(SICI)1522-7219(200003)9:1<1::AID-ICD210>3.0.CO;2-V

[ref110] Hudgins C. V. Numbers F. C. (1942). An investigation of the intelligibility of the speech of the deaf. Genet. Psychol. Monogr.

[ref004] Imafuku M. Kanakogi Y. Butler D. Myowa M. (2019). Demystifying infant vocal imitation: the roles of mouth looking and speaker’s gaze. Dev. Sci. 22:e12825. doi: 10.1111/desc.12825 30980494

[ref111] Jang H. Ha S. Jang H. Ha S. (2019). Protophone development at 4-6 months and 7-9 months of age. Commun. Sci. Disorders 24, 707–714. doi: 10.12963/csd.19641

[ref112] Janik V. M. Slater P. J. (2000). The different roles of social learning in vocal communication. Anim. Behav. 60, 1–11. doi: 10.1006/anbe.2000.1410, PMID: 10924198

[ref113] Jarvis E. D. (2004). Learned birdsong and the neurobiology of human language. Ann. N. Y. Acad. Sci. 1016, 749–777. doi: 10.1196/annals.1298.038, PMID: 15313804 PMC2485240

[ref114] Jellinger K. (1990). New developments in the pathology of Parkinson's disease. Adv. Neurol. 53, 1–16. 1978509

[ref115] Jin X. Costa R. M. (2015). Shaping action sequences in basal ganglia circuits. Curr. Opin. Neurobiol. 33, 188–196. doi: 10.1016/j.conb.2015.06.011, PMID: 26189204 PMC4523429

[ref116] Jones J. A. Munhall K. G. (2005). Remapping auditory-motor representations in voice production. Curr. Biol. 15, 1768–1772. doi: 10.1016/j.cub.2005.08.063, PMID: 16213825

[ref117] Jusczyk P. W. (1997). The Discovery of Spoken Language. Cambridge, MA: MIT Press.

[ref118] Jusczyk P. W. Friederici A. D. Wessels J. M. Svenkerud V. Y. Jusczyk A. M. (1993). Infants′ sensitivity to the sound patterns of native language words. J. Mem. Lang. 32, 402–420. doi: 10.1006/jmla.1993.1022

[ref119] Katseff S. Houde J. Johnson K. (2012). Partial compensation for altered auditory feedback: a tradeoff with somatosensory feedback? Lang. Speech 55, 295–308. doi: 10.1177/0023830911417802, PMID: 22783636

[ref120] Kawahara H. (1994). “Effects of natural auditory feedback on fundamental frequency control.” in *Third international conference on spoken language processing*.

[ref121] Kearney M. G. (2020). An actor-critic circuit in the songbird enables vocal learning. Doctoral dissertation. Duke University.

[ref122] Kent R. D. Murray A. D. (1982). Acoustic features of infant vocalic utterances at 3, 6, and 9 months. J. Acoust. Soc. Am. 72, 353–365. doi: 10.1121/1.388089, PMID: 7119278

[ref123] Klaus A. Alves da Silva J. Costa R. M. (2019). What, if, and when to move: basal ganglia circuits and self-paced action initiation. Annu. Rev. Neurosci. 42, 459–483. doi: 10.1146/annurev-neuro-072116-031033, PMID: 31018098

[ref005] Kokkinaki T. Kugiumutzakis G. (2000). Basic aspects of vocal imitation in infant-parent interaction during the first 6 months. J. Reprod. Infant. Psychol. 18, 173–187. doi: 10.1080/713683042

[ref124] Konda V. R. Tsitsiklis J. N. (2003). On actor-critic algorithms. SIAM J. Control. Optim. 42, 1143–1166. doi: 10.1137/S0363012901385691

[ref125] Konishi M. (1963a). The role of auditory feedback in the vocal behavior of the domestic fowl 1. Z. Tierpsychol. 20, 349–367.

[ref126] Konishi M. (1963b). The role of audition in the development and maintenance of avian vocal behavior. PhD thesis. University of California, Berkeley.

[ref127] Konishi M. (1964). Effects of deafening on song development in two species of juncos. Condor 66, 85–102. doi: 10.2307/1365388

[ref128] Konishi M. (1965a). The role of auditory feedback in the control of vocalization in the white-crowned sparrow 1. Z. Tierpsychol. 22, 770–783. doi: 10.1111/j.1439-0310.1965.tb01688.x, PMID: 5874921

[ref129] Konishi M. (1965b). Effects of deafening on song development in American robins and black-headed grosbeaks. Z. Tierpsychol. 5879978

[ref130] Konishi M. (1985). Birdsong: from behavior to neuron. Annu. Rev. Neurosci. 8, 125–170. doi: 10.1146/annurev.ne.08.030185.001013 3885827

[ref131] Konishi M. (2010). From central pattern generator to sensory template in the evolution of birdsong. Brain Lang. 115, 18–20. doi: 10.1016/j.bandl.2010.05.001, PMID: 20955898

[ref132] Koopmans-van Beinum F. J. Stelt J. M. (1986). “Early stages in the development of speech movements,” in Precursors of Early Speech. eds. Lindblom B. Zetterstrom R. (London: Palgrave Macmillan), 37–50.

[ref133] Krakauer J. W. Hadjiosif A. M. Xu J. Wong A. L. Haith A. M. (2019). Motor learning. Compr. Physiol. 9, 613–663. doi: 10.1002/cphy.c170043, PMID: 30873583

[ref134] Krishnan A. Xu Y. Gandour J. Cariani P. (2005). Encoding of pitch in the human brainstem is sensitive to language experience. Cogn. Brain Res. 25, 161–168. doi: 10.1016/j.cogbrainres.2005.05.004, PMID: 15935624

[ref006] Kroodsma D. E. Konishi M. (1991). A suboscine bird (eastern phoebe, Sayornis phoebe) develops normal song without auditory feedback. Anim. Behav. 42, 477–487. doi: 10.1016/S0003-3472(05)80047-8

[ref135] Kugiumutzakis G. (1999). “Genesis and development of early infant mimesis to facial and vocal models,” in Imitation in Infancy. eds. Nadel J. Butterworth G. (Cambridge University Press), 36–59.

[ref136] Kuhl P. K. (1992). Infants’ perception and representation of speech: development of a new theory. in “Proceedings of the international conference on spoken language processing.” (eds.) J. Ohala, T. M. Nearey, B. L. Derwing, M. M. Hodge, and G. E. Wiebe; University of Alberta Press, 449–456.

[ref137] Kuhl P. K. (2000). A new view of language acquisition. Proc. Natl. Acad. Sci. 97, 11850–11857. doi: 10.1073/pnas.97.22.11850, PMID: 11050219 PMC34178

[ref138] Kuhl P. K. (2003). Human speech and birdsong: communication and the social brain. Proc. Natl. Acad. Sci. 100, 9645–9646. doi: 10.1073/pnas.1733998100, PMID: 12913121 PMC187796

[ref139] Kuhl P. K. Andruski J. E. Chistovich I. A. Chistovich L. A. Kozhevnikova E. V. Ryskina V. L.. (1997). Cross-language analysis of phonetic units in language addressed to infants. Science 277, 684–686. doi: 10.1126/science.277.5326.684, PMID: 9235890

[ref140] Kuhl P. K. Meltzoff A. N. (1996). Infant vocalizations in response to speech: vocal imitation and developmental change. J. Acoust. Soc. Am. 100, 2425–2438. doi: 10.1121/1.417951, PMID: 8865648 PMC3651031

[ref141] Kuhl P. K. Stevens E. Hayashi A. Deguchi T. Kiritani S. Iverson P. (2006). Infants show a facilitation effect for native language phonetic perception between 6 and 12 months. Dev. Sci. 9, F13–F21. doi: 10.1111/j.1467-7687.2006.00468.x, PMID: 16472309

[ref142] Kuhl P. K. Williams K. A. Lacerda F. Stevens K. N. Lindblom B. (1992). Linguistic experience alters phonetic perception in infants by 6 months of age. Science 255, 606–608. doi: 10.1126/science.1736364, PMID: 1736364

[ref143] Ladefoged P. (1996). Elements of Acoustic Phonetics University of Chicago Press.

[ref144] Larson C. R. Altman K. W. Liu H. Hain T. C. (2008). Interactions between auditory and somatosensory feedback for voice F 0 control. Exp. Brain Res. 187, 613–621. doi: 10.1007/s00221-008-1330-z, PMID: 18340440 PMC2763543

[ref145] Leblois A. Wendel B. J. Perkel D. J. (2010). Striatal dopamine modulates basal ganglia output and regulates social context-dependent behavioral variability through D1 receptors. J. Neurosci. 30, 5730–5743. doi: 10.1523/JNEUROSCI.5974-09.2010, PMID: 20410125 PMC2866011

[ref146] Lee S. A. S. Davis B. MacNeilage P. (2010). Universal production patterns and ambient language influences in babbling: a cross-linguistic study of Korean-and English-learning infants. J. Child Lang. 37, 293–318. doi: 10.1017/S0305000909009532, PMID: 19570317

[ref147] Lester B. M. Boukydis C. Z. (in press). “No language but a cry,” in Nonverbal Vocal Communication: Comparative and Developmental Approaches. eds. Papougek H. Jiur- gens U. Papougek M. (Cambridge: Cambridge University Press), 145–173.

[ref148] Levelt W. J. (1993). Speaking: From Intention to Articulation MIT press.

[ref149] Levitt A. G. Utman J. G. A. (1992). From babbling toward the sound systems of English and French: a longitudinal two-case study. J. Child Lang. 19, 19–49. doi: 10.1017/S0305000900013611, PMID: 1551932

[ref150] Levitt A. G. Wang Q. (1991). Evidence for language-specific rhythmic influences in the reduplicative babbling of French-and English-learning infants. Lang. Speech 34, 235–249. doi: 10.1177/002383099103400302 1843525

[ref151] Lieberman P. (2000). Human Language and Our Reptilian Brain: The Subcortical Bases of Speech, Syntax, and Thought. Cambridge, MA: Harvard University Press 10.1353/pbm.2001.0011 11253303

[ref152] Lieberman P. (2012). Vocal tract anatomy and the neural bases of talking. J. Phon. 40, 608–622. doi: 10.1016/j.wocn.2012.04.001

[ref153] Lieberman P. Crelin E. S. Klatt D. H. (1972). Phonetic ability and related anatomy of the newborn and adult human, Neanderthal man, and the chimpanzee. Am. Anthropol. 74, 287–307. doi: 10.1525/aa.1972.74.3.02a00020

[ref154] Lieberman P. Kako E. Friedman J. Tajchman G. Feldman L. S. Jiminez E. B. (1992). Speech production, syntax comprehension, and cognitive deficits in Parkinson's disease. Brain Lang. 43, 169–189. doi: 10.1016/0093-934X(92)90127-Z, PMID: 1393519

[ref155] Lieberman P. McCarthy R. C. (2015). “The evolution of speech and language,” in Handbook of Paleoanthropology. eds. Henke W. Tattersall I. (Heidelberg: Springer Berlin), 873–920.

[ref156] Lieberman D. E. McCarthy R. C. Hiiemae K. M. Palmer J. B. (2001). Ontogeny of postnatal hyoid and larynx descent in humans. Arch. Oral Biol. 46, 117–128. doi: 10.1016/S0003-9969(00)00108-4, PMID: 11163319

[ref157] Liljencrants J. Lindblom B. (1972). Numerical simulation of vowel quality systems: the role of perceptual contrast. Language 48, 839–862. doi: 10.2307/411991

[ref158] Lindblom B. (2000). Developmental origins of adult phonology: the interplay between phonetic emergents and the evolutionary adaptations of sound patterns. Phonetica 57, 297–314. doi: 10.1159/000028482, PMID: 10992149

[ref159] Lindblom B. Lubker J. Gay T. (1979). Formant frequencies of some fixed-mandible vowels and a model of speech motor programming by predictive simulation. J. Phon. 7, 147–161. doi: 10.1016/S0095-4470(19)31046-0

[ref160] Lindblom B. Maddieson I. (1988). “Phonetic universals in consonant systems,” in Language, Speech and Mind. eds. Hyman L. M. Li C. N. (Routledge).

[ref161] Lindblom B. Sundberg J. (1969). A quantitative model of vowel production and the distinctive features of Swedish vowels. Q. Progress Status Rep. Speech Trans. Lab. Roy. Instit. Technol. 10, 14–30.

[ref162] Locke J. L. (1993). The Child’s Path to Spoken Language Harvard University Press.

[ref163] Locke J. L. Pearson D. M. (1992). “Vocal learning and the emergence of phonological capacity: A neurobiological approach,” in Phonological Development: Models, Research, Implications. eds. Ferguson C. A. Menn L. Stoel-Gammon C., (York, Timonium: MD), 91–129.

[ref164] Locke J. L. Snow C. (2010). “Social influences on vocal learning in human and nonhumanprimates,” in Social Influences on Vocal Development. eds. Snowdon C. T. Hausberger M. (Cambridge University Press), 274–293.

[ref165] Lund J. P. Kolta A. (2006). Brainstem circuits that control mastication: do they have anything to say during speech? J. Commun. Disord. 39, 381–390. doi: 10.1016/j.jcomdis.2006.06.014, PMID: 16884732

[ref166] Ma X. Suga N. (2003). Augmentation of plasticity of the central auditory system by the basal forebrain and/or somatosensory cortex. J. Neurophysiol. 89, 90–103. doi: 10.1152/jn.00968.2001 12522162

[ref167] MacNeilage P. F. (1998). The frame/content theory of evolution of speech production. Behav. Brain Sci. 21, 499–511. doi: 10.1017/S0140525X98001265, PMID: 10097020

[ref168] MacNeilage P. F. Davis B. L. (2000). Deriving speech from nonspeech: a view from ontogeny. Phonetica 57, 284–296. doi: 10.1159/000028481, PMID: 10992148

[ref170] Maddieson I. (1984). Patterns of Sounds Cambridge university press.

[ref171] Maguire G. A. Nguyen D. L. Simonson K. C. Kurz T. L. (2020). The pharmacologic treatment of stuttering and its neuropharmacologic basis. Front. Neurosci. 14:158. doi: 10.3389/fnins.2020.00158, PMID: 32292321 PMC7118465

[ref172] Maguire G. A. Yeh C. Y. Ito B. S. (2012). Overview of the diagnosis and treatment of stuttering. J. Exper. Clin. Med. 4, 92–97. doi: 10.1016/j.jecm.2012.02.001

[ref007] Malenka R. C. Bear M. F. (2004). LTP and LTD: an embarrassment of riches. Neuron 44, 5–21. doi: 10.1016/j.neuron.2004.09.012 15450156

[ref173] Mampe B. Friederici A. D. Christophe A. Wermke K. (2009). Newborns' cry melody is shaped by their native language. Curr. Biol. 19, 1994–1997. doi: 10.1016/j.cub.2009.09.064, PMID: 19896378

[ref174] Marler P. (1970). Birdsong and speech development: could there be parallels? There may be basic rules governing vocal learning to which many species conform, including man. Am. Sci. 58, 669–673. 5480089

[ref175] Marler P. Waser M. S. (1977). Role of auditory feedback in canary song development. J. Comp. Physiol. Psychol. 91, 8–16. doi: 10.1037/h0077303, PMID: 838918

[ref176] Marsden C. D. Obeso J. A. (1994). The functions of the basal ganglia and the paradox of stereotaxic surgery in Parkinson's disease. Brain 117, 877–897. doi: 10.1093/brain/117.4.877, PMID: 7922472

[ref177] Marshall P. J. Meltzoff A. N. (2014). Neural mirroring mechanisms and imitation in human infants. Philos. Trans. Roy. Soc. B. Biol. Sci. 369:20130620. doi: 10.1098/rstb.2013.0620, PMID: 24778387 PMC4006193

[ref179] McCarthy D. (1946). “Language development in children,” in Manual of Child Psychology. ed. Carmichael L.. 2nd *Edn*. (New York: John Wiley & Sons, Inc.).

[ref180] Meltzoff A. N. Moore M. K. (1989). Imitation in newborn infants: exploring the range of gestures imitated and the underlying mechanisms. Dev. Psychol. 25, 954–962. doi: 10.1037/0012-1649.25.6.954, PMID: 25147405 PMC4137867

[ref181] Messum P. R. (2008). The Role of Imitation in Learning to Pronounce. University College London (United Kingdom): University of London

[ref182] Mesulam M. M. (1990). Large-scale neurocognitive networks and distributed processing for attention, language, and memory. Ann. Neurol. 28, 597–613. doi: 10.1002/ana.410280502, PMID: 2260847

[ref183] Metfessel M. (1935). Roller canary song produced without learning from external sources. Science 81:470. doi: 10.1126/science.81.2106.470.a, PMID: 17818769

[ref184] Middleton F. A. Strick P. L. (2002). Basal-ganglia ‘projections’ to the prefrontal cortex of the primate. Cereb. Cortex 12, 926–935. doi: 10.1093/cercor/12.9.926, PMID: 12183392

[ref185] Moayedi Y. Michlig S. Park M. Koch A. Lumpkin E. A. (2021). Somatosensory innervation of healthy human oral tissues. J. Comp. Neurol. 529, 3046–3061. doi: 10.1002/cne.25148, PMID: 33786834 PMC10052750

[ref186] Molina-Luna K. Pekanovic A. Röhrich S. Hertler B. Schubring-Giese M. Rioult-Pedotti M. S.. (2009). Dopamine in motor cortex is necessary for skill learning and synaptic plasticity. PLoS One 4:e7082. doi: 10.1371/journal.pone.0007082, PMID: 19759902 PMC2738964

[ref187] Montag C. Bleek B. Faber J. Reuter M. (2012). The role of the DRD2 C957T polymorphism in neuroticism in persons who stutter and healthy controls. Neuroreport 23, 246–250. doi: 10.1097/WNR.0b013e3283505b8a, PMID: 22262089

[ref188] Murdoch B. E. (2001). Subcortical brain mechanisms in speech and language. Folia Phoniatr. Logop. 53, 233–251. doi: 10.1159/000052679 11464066

[ref189] Murdoch B. E. (2009). Speech and Language Disorders Associated With Subcortical Pathology John Wiley & Sons.

[ref191] Nathani S. Ertmer D. J. Stark R. E. (2006). Assessing vocal development in infants and toddlers. Clin. Linguist. Phonet. 20, 351–369. doi: 10.1080/02699200500211451, PMID: 16728333 PMC3412408

[ref192] Negus V. (1949). The Comparative Anatomy and Physiology of the Larynx, Heinemann.

[ref193] Nishimura T. (2018). The descended larynx and the descending larynx. Anthropol. Sci. 126, 3–8. doi: 10.1537/ase.180301

[ref194] Noad M. J. Cato D. H. Bryden M. M. Jenner M. N. Jenner K. C. S. (2000). Cultural revolution in whale songs. Nature 408:537. doi: 10.1038/35046199, PMID: 11117730

[ref195] Nottebohm F. (1970). Ontogeny of bird song: different strategies in vocal development are reflected in learning stages, critical periods, and neural lateralization. Science 167, 950–956. doi: 10.1126/science.167.3920.950 17749614

[ref196] Nottebohm F. Nottebohm M. E. Crane L. (1986). Developmental and seasonal changes in canary song and their relation to changes in the anatomy of song-control nuclei. Behav. Neural Biol. 46, 445–471. doi: 10.1016/S0163-1047(86)90485-1, PMID: 3814048

[ref197] Nudel R. Newbury D. F. (2013). Foxp2. Wiley Interdiscip. Rev. Cogn. Sci. 4, 547–560. doi: 10.1002/wcs.1247, PMID: 24765219 PMC3992897

[ref198] Ochs E. Schieffelin B. (2009). “Language acquisition and socialization: Three developmental stories and their implications,” Linguistic Anthropology: A reader, *2nd edn*, 296–328.

[ref199] Olkowicz S. Kocourek M. Lučan R. K. Porteš M. Fitch W. T. Herculano-Houzel S.. (2016). Birds have primate-like numbers of neurons in the forebrain. Proc. Natl. Acad. Sci. 113, 7255–7260. doi: 10.1073/pnas.1517131113, PMID: 27298365 PMC4932926

[ref200] Oller D. K. (1980). “The ergence of the sounds of speech in infancy” in Child Phonology, Volume 1: Production. eds. Yeni-Komshian G. Kavanagh J. Ferguson C. (New York, NY: Academic Press), 93–112.

[ref201] Oller D. K. (2000). The Emergence of the Speech Capacity Psychology Press.

[ref202] Oller D. K. Buder E. H. Ramsdell H. L. Warlaumont A. S. Chorna L. Bakeman R. (2013). Functional flexibility of infant vocalization and the emergence of language. Proc. Natl. Acad. Sci. 110, 6318–6323. doi: 10.1073/pnas.1300337110, PMID: 23550164 PMC3631625

[ref203] Oller D. K. Eilers R. E. (1988). The role of audition in infant babbling. Child Dev. 59, 441–449. doi: 10.2307/1130323, PMID: 3359864

[ref204] Oller D. K. Ramsay G. Bene E. Long H. L. Griebel U. (2021). Protophones, the precursors to speech, dominate the human infant vocal landscape. Philos. Trans. R. Soc. B 376:20200255. doi: 10.1098/rstb.2020.0255, PMID: 34482735 PMC8419580

[ref205] Papoutsi M. de Zwart J. A. Jansma J. M. Pickering M. J. Bednar J. A. Horwitz B. (2009). From phonemes to articulatory codes: an fMRI study of the role of Broca's area in speech production. Cereb. Cortex 19, 2156–2165. doi: 10.1093/cercor/bhn239, PMID: 19181696 PMC2722428

[ref206] Paulin M. G. (1993). The role of the cerebellum in motor control and perception. Brain Behav. Evol. 41, 39–50. doi: 10.1159/000113822 8431754

[ref207] Penfield W. (1954). Mechanisms of voluntary movement. Brain 77, 1–17. doi: 10.1093/brain/77.1.1 13160255

[ref208] Penfield W. Boldrey E. (1937). Somatic motor and sensory representation in the cerebral cortex of man as studied by electrical stimulation. Brain 60, 389–443. doi: 10.1093/brain/60.4.389

[ref209] Pepperberg I. M. (2010). Vocal learning in Grey parrots: a brief review of perception, production, and cross-species comparisons. Brain Lang. 115, 81–91. doi: 10.1016/j.bandl.2009.11.002, PMID: 20199805

[ref210] Perkins W. H. Kent R. D. Curlee R. F. (1991). A theory of neuropsycholinguistic function in stuttering. J. Speech Lang. Hear. Res. 34, 734–752. doi: 10.1044/jshr.3404.734, PMID: 1956181

[ref211] Person A. L. Gale S. D. Farries M. A. Perkel D. J. (2008). Organization of the songbird basal ganglia, including area X. J. Comp. Neurol. 508, 840–866. doi: 10.1002/cne.21699, PMID: 18398825

[ref212] Perszyk D. R. Waxman S. R. (2019). Infants’ advances in speech perception shape their earliest links between language and cognition. Sci. Rep. 9, 1–6. doi: 10.1038/s41598-019-39511-9 30824848 PMC6397155

[ref213] Petkov C. I. Jarvis E. (2012). Birds, primates, and spoken language origins: behavioral phenotypes and neurobiological substrates. Front. Evol. Neurosci. 4:12. doi: 10.3389/fnevo.2012.00012 22912615 PMC3419981

[ref214] Pickett E. R. Kuniholm E. Protopapas A. Friedman J. Lieberman P. (1998). Selective speech motor, syntax and cognitive deficits associated with bilateral damage to the putamen and the head of the caudate nucleus: a case study. Neuropsychologia 36, 173–188. doi: 10.1016/S0028-3932(97)00065-1, PMID: 9539237

[ref215] Pidoux L. Le Blanc P. Levenes C. Leblois A. (2018). A subcortical circuit linking the cerebellum to the basal ganglia engaged in vocal learning. elife 7:e32167. doi: 10.7554/eLife.32167, PMID: 30044222 PMC6112851

[ref216] Pile E. J. Dajani H. R. Purcell D. W. Munhall K. G. (2007). “Talking under conditions of altered auditory feedback: Does adaptation of one vowel generalize to other vowels?” in Proceedings of the 16th International Congress of Phonetic Sciences (ICPhS). Saarbrücken, Germany, 645–648.

[ref217] Pinker S. Bloom P. (1990). Natural language and natural selection. Behav. Brain Sci. 13, 707–727. doi: 10.1017/S0140525X00081061

[ref218] Poulsen H. (1959). Song learning in the domestic canary. Z. Tierpsychol. 16, 173–178. doi: 10.1111/j.1439-0310.1959.tb02052.x

[ref219] Poulson C. L. Kymissis E. Reeve K. F. Andreatos M. Reeve L. (1991). Generalized vocal imitation in infants. J. Exp. Child Psychol. 51, 267–279. doi: 10.1016/0022-0965(91)90036-R, PMID: 2033363

[ref220] Prather J. F. Okanoya K. Bolhuis J. J. (2017). Brains for birds and babies: neural parallels between birdsong and speech acquisition. Neurosci. Biobehav. Rev. 81, 225–237. doi: 10.1016/j.neubiorev.2016.12.035, PMID: 28087242

[ref221] Price P. H. (1979). Developmental determinants of structure in zebra finch song. J. Comp. Physiol. Psychol. 93, 260–277. doi: 10.1037/h0077553

[ref222] Purcell D. W. Munhall K. G. (2006). Adaptive control of vowel formant frequency: evidence from real-time formant manipulation. J. Acoust. Soc. Am. 120, 966–977. doi: 10.1121/1.2217714, PMID: 16938984

[ref223] Radanovic M. Scaff M. (2003). Speech and language disturbances due to subcortical lesions. Brain Lang. 84, 337–352. doi: 10.1016/S0093-934X(02)00554-0, PMID: 12662975

[ref224] Rauschecker J. P. (2012). Ventral and dorsal streams in the evolution of speech and language. Front. Evol. Neurosci. 4:7. doi: 10.3389/fnevo.2012.00007 22615693 PMC3351753

[ref225] Rauschecker J. P. (2018). Where did language come from? Precursor mechanisms in nonhuman primates. Curr. Opin. Behav. Sci. 21, 195–204. doi: 10.1016/j.cobeha.2018.06.003, PMID: 30778394 PMC6377164

[ref226] Rauschecker J. P. Scott S. K. (2009). Maps and streams in the auditory cortex: nonhuman primates illuminate human speech processing. Nat. Neurosci. 12, 718–724. doi: 10.1038/nn.2331, PMID: 19471271 PMC2846110

[ref227] Reichmuth C. Casey C. (2014). Vocal learning in seals, sea lions, and walruses. Curr. Opin. Neurobiol. 28, 66–71. doi: 10.1016/j.conb.2014.06.011, PMID: 25042930

[ref228] Reimers-Kipping S. Hevers W. Pääbo S. Enard W. (2011). Humanized Foxp2 specifically affects cortico-basal ganglia circuits. Neuroscience 175, 75–84. doi: 10.1016/j.neuroscience.2010.11.042, PMID: 21111790

[ref229] Reiner A. Perkel D. J. Mello C. V. Jarvis E. D. (2004). Songbirds and the revised avian brain nomenclature. Ann. N. Y. Acad. Sci. 1016, 77–108. doi: 10.1196/annals.1298.013, PMID: 15313771 PMC2481519

[ref008] Rosenberger P. B. Wheelden J. A. Kalotkin M. (1976). The effect of haloperidol on stuttering. Am. J. Psychiatry. 133, 331–334. doi: 10.1176/ajp.133.3.331 1259046

[ref230] Ruben R. J. (1997). A time frame of critical/sensitive periods of language development. Acta Otolaryngol. 117, 202–205. doi: 10.3109/00016489709117769, PMID: 9105448

[ref231] Sanes D. H. Bao S. (2009). Tuning up the developing auditory CNS. Curr. Opin. Neurobiol. 19, 188–199. doi: 10.1016/j.conb.2009.05.014, PMID: 19535241 PMC2717554

[ref232] Sapir S. Baker K. K. Larson C. R. Ramig L. O. (2000). Short-latency changes in voice F0 and neck surface EMG induced by mechanical perturbations of the larynx during sustained vowel phonation. J. Speech Lang. Hear. Res. 43, 268–276. doi: 10.1044/jslhr.4301.268, PMID: 10668668

[ref234] Schroeder C. E. Lindsley R. W. Specht C. Marcovici A. Smiley J. F. Javitt D. C. (2001). Somatosensory input to auditory association cortex in the macaque monkey. J. Neurophysiol. 85, 1322–1327. doi: 10.1152/jn.2001.85.3.1322, PMID: 11248001

[ref235] Schultz W. (2007). Behavioral dopamine signals. Trends Neurosci. 30, 203–210. doi: 10.1016/j.tins.2007.03.007 17400301

[ref236] Schultz W. (2013). Updating dopamine reward signals. Curr. Opin. Neurobiol. 23, 229–238. doi: 10.1016/j.conb.2012.11.012, PMID: 23267662 PMC3866681

[ref237] Schusterman R. J. (2008). “Vocal learning in mammals with special emphasis on pinnipeds,” in The Evolution of Communicative Flexibility: Complexity, Creativity, and Adaptability in Human and Animal Communication. eds. Oller D. K. Gribel U. (Cambridge, MA: MIT Press), 41–70.

[ref238] Shiba K. Yoshida K. Nakajima Y. Konno A. (1997). Influences of laryngeal afferent inputs on intralaryngeal muscle activity during vocalization in the cat. Neurosci. Res. 27, 85–92. doi: 10.1016/S0168-0102(96)01136-4, PMID: 9089702

[ref239] Simonyan K. Horwitz B. (2011). Laryngeal motor cortex and control of speech in humans. Neuroscientist 17, 197–208. doi: 10.1177/1073858410386727, PMID: 21362688 PMC3077440

[ref240] Simonyan K. Horwitz B. Jarvis E. D. (2012). Dopamine regulation of human speech and bird song: a critical review. Brain Lang. 122, 142–150. doi: 10.1016/j.bandl.2011.12.009, PMID: 22284300 PMC3362661

[ref241] Smeets W. J. Marin O. Gonzalez A. (2000). Evolution of the basal ganglia: new perspectives through a comparative approach. J. Anatomy 196, 501–517. doi: 10.1046/j.1469-7580.2000.19640501.x, PMID: 10923983 PMC1468093

[ref242] Smith C. R. (1975). Residual hearing and speech production in deaf children. J. Speech Hear. Res. 18, 795–811. doi: 10.1044/jshr.1804.795, PMID: 1207108

[ref243] Sober S. J. Wohlgemuth M. J. Brainard M. S. (2008). Central contributions to acoustic variation in birdsong. J. Neurosci. 28, 10370–10379. doi: 10.1523/JNEUROSCI.2448-08.2008, PMID: 18842896 PMC2613831

[ref244] Stark R. E. (1980). “Stages of speech development in the first year of life,” in Child Phonology. eds. Yeni-Komshian G. Kavanagh J. Ferguson C., vol. 1 (Academic Press), 73–92.

[ref245] Stephenson-Jones M. Kardamakis A. A. Robertson B. Grillner S. (2013). Independent circuits in the basal ganglia for the evaluation and selection of actions. Proc. Natl. Acad. Sci. 110, E3670–E3679. doi: 10.1073/pnas.1314815110 24003130 PMC3780871

[ref246] Stevens K. N. (1972). “The quantal nature of speech: evidence from articulatory-acoustic data,” in Human Communication: A Unified View. eds. David E. E. Jr. Denes P. B. (New York: McGraw–Hill), 51–66.

[ref247] Stevens K. N. (1989). On the quantal nature of speech. J. Phon. 17, 3–45. doi: 10.1016/S0095-4470(19)31520-7

[ref248] Stevens K. N. (2000). Acoustic Phonetics, *Vol*. 30, MIT press.

[ref249] Stuss D. T. Benson D. F. Clermont R. Della Malva C. L. Kaplan E. F. Weir W. S. (1986). Language functioning after bilateral prefrontal leukotomy. Brain Lang. 28, 66–70. doi: 10.1016/0093-934X(86)90091-X, PMID: 2424546

[ref250] Suthers R. A. (1997). Peripheral control and lateralization of birdsong. J. Neurobiol. 33, 632–652. doi: 10.1002/(SICI)1097-4695(19971105)33:5<632::AID-NEU10>3.0.CO;2-B, PMID: 9369464

[ref251] Tepper J. M. Abercrombie E. D. Bolam J. P. (2007). Basal ganglia macrocircuits. Prog. Brain Res. 160, 3–7. doi: 10.1016/S0079-6123(06)60001-0, PMID: 17499105

[ref252] Tourville J. A. Reilly K. J. Guenther F. H. (2008). Neural mechanisms underlying auditory feedback control of speech. NeuroImage 39, 1429–1443. doi: 10.1016/j.neuroimage.2007.09.054, PMID: 18035557 PMC3658624

[ref253] Tumer E. C. Brainard M. S. (2007). Performance variability enables adaptive plasticity of ‘crystallized’ adult birdsong. Nature 450, 1240–1244. doi: 10.1038/nature06390, PMID: 18097411

[ref254] Ullman M. T. (2001). A neurocognitive perspective on language: the declarative/procedural model. Nat. Rev. Neurosci. 2, 717–726. doi: 10.1038/35094573, PMID: 11584309

[ref255] Vallabha G. K. McClelland J. L. Pons F. Werker J. F. Amano S. (2007). Unsupervised learning of vowel categories from infant-directed speech. Proc. Natl. Acad. Sci. 104, 13273–13278. doi: 10.1073/pnas.0705369104, PMID: 17664424 PMC1934922

[ref256] Vaughn K. A. Hernandez A. E. (2018). Becoming a balanced, proficient bilingual: predictions from age of acquisition & genetic background. J. Neurolinguistics 46, 69–77. doi: 10.1016/j.jneuroling.2017.12.012, PMID: 30038460 PMC6054315

[ref257] Vaughn K. A. Nuñez A. I. R. Greene M. R. Munson B. A. Grigorenko E. L. Hernandez A. E. (2016). Individual differences in the bilingual brain: the role of language background and DRD2 genotype in verbal and non-verbal cognitive control. J. Neurolinguistics 40, 112–127. doi: 10.1016/j.jneuroling.2016.06.008, PMID: 28082765 PMC5222542

[ref258] Vernes S. C. Wilkinson G. S. (2020). Behavior, biology and evolution of vocal learning in bats. Philos. Trans. R. Soc. B 375:20190061. doi: 10.1098/rstb.2019.0061, PMID: 31735153 PMC6895559

[ref259] Vihman M. M. (2013). Phonological development: The First Two Years John Wiley & Sons.

[ref260] Vihman M. M. (2019). Phonological Templates in Development Oxford University Press.

[ref261] Wang X. Honda K. Dang J. Wang H. Wei J. (2015b). “Influences of auditory and vibrotactile information on vocal F0 responses.” in *2015 Asia-Pacific signal and information processing association annual summit and conference (APSIPA)* (pp. 160–164). IEEE.

[ref262] Wang X. Honda K. Dang J. Wei J. (2015a). “Vocal responses to frequency modulated composite sinewaves via auditory and vibrotactile pathways.” in *2015 IEEE international conference on acoustics, speech and signal processing (ICASSP)*. IEEE, 4355–4359.

[ref263] Warren T. L. Tumer E. C. Charlesworth J. D. Brainard M. S. (2011). Mechanisms and time course of vocal learning and consolidation in the adult songbird. J. Neurophysiol. 106, 1806–1821. doi: 10.1152/jn.00311.2011, PMID: 21734110 PMC3191835

[ref264] Werker J. F. Tees R. C. (1984). Cross-language speech perception: evidence for perceptual reorganization during the first year of life. Infant Behav. Dev. 7, 49–63. doi: 10.1016/S0163-6383(84)80022-3

[ref265] Wermke K. Robb M. P. Schluter P. J. (2021). Melody complexity of infants’ cry and non-cry vocalisations increases across the first six months. Sci. Rep. 11, 1–11. doi: 10.1038/s41598-021-83564-8 33602997 PMC7893022

[ref266] Wich S. A. Swartz K. B. Hardus M. E. Lameira A. R. Stromberg E. Shumaker R. W. (2009). A case of spontaneous acquisition of a human sound by an orangutan. Primates 50, 56–64. doi: 10.1007/s10329-008-0117-y, PMID: 19052691

[ref267] Wickens J. R. Reynolds J. N. Hyland B. I. (2003). Neural mechanisms of reward-related motor learning. Curr. Opin. Neurobiol. 13, 685–690. doi: 10.1016/j.conb.2003.10.013, PMID: 14662369

[ref268] Wildgruber D. Ackermann H. Grodd W. (2001). Differential contributions of motor cortex, basal ganglia, and cerebellum to speech motor control: effects of syllable repetition rate evaluated by fMRI. NeuroImage 13, 101–109. doi: 10.1006/nimg.2000.0672, PMID: 11133313

[ref269] Wildgruber D. Ackermann H. Klose U. Kardatzki B. Grodd W. (1996). Functional lateralization of speech production at primary motor cortex: a fMRI study. Neuroreport 7, 2791–2796. doi: 10.1097/00001756-199611040-00077, PMID: 8981469

[ref270] Williams G. V. Goldman-Rakic P. S. (1995). Modulation of memory fields by dopamine dl receptors in prefrontal cortex. Nature 376, 572–575. doi: 10.1038/376572a0, PMID: 7637804

[ref271] Wise R. A. (2004). Dopamine, learning and motivation. Nat. Rev. Neurosci. 5, 483–494. doi: 10.1038/nrn1406 15152198

[ref272] Wong P. C. Morgan-Short K. Ettlinger M. Zheng J. (2012). Linking neurogenetics and individual differences in language learning: the dopamine hypothesis. Cortex 48, 1091–1102. doi: 10.1016/j.cortex.2012.03.017, PMID: 22565204 PMC3965203

[ref273] Wong P. C. Perrachione T. K. Gunasekera G. Chandrasekaran B. (2009). Communication disorders in speakers of tone languages: etiological bases and clinical considerations in Seminars in speech and language (*Vol. 30*, No. 03). Thieme Medical Publishers, 162–173. 10.1055/s-0029-1225953 PMC2805066 19711234

[ref274] Wong P. Strange W. (2017). Phonetic complexity affects children’s mandarin tone production accuracy in disyllabic words: a perceptual study. PLoS One 12:e0182337. doi: 10.1371/journal.pone.0182337, PMID: 28806417 PMC5555563

[ref275] Wood A. N. (2021). New roles for dopamine in motor skill acquisition: lessons from primates, rodents, and songbirds. J. Neurophysiol. 125, 2361–2374. doi: 10.1152/jn.00648.2020, PMID: 33978497 PMC8285659

[ref276] Wu J. C. Maguire G. Riley G. Lee A. Keator D. Tang C.. (1997). Increased dopamine activity associated with stuttering. Neuroreport 8, 767–770. doi: 10.1097/00001756-199702100-00037, PMID: 9106763

[ref277] Yairi E. Ambrose N. (2013). Epidemiology of stuttering: 21st century advances. J. Fluen. Disord. 38, 66–87. doi: 10.1016/j.jfludis.2012.11.002, PMID: 23773662 PMC3687212

[ref278] Yanagihara S. Yazaki-Sugiyama Y. (2016). Auditory experience-dependent cortical circuit shaping for memory formation in bird song learning. Nat. Commun. 7, 1–11. doi: 10.1038/ncomms11946 PMC4919517 27327620

